# Supplementary figures and images for: Emotion regulation success involves systematic gradient-based reconfigurations of large-scale activation patterns in the human brain
Source: PLoS Biol. 2026 Apr 2;24(4):e3003666. doi: 10.1371/journal.pbio.3003666 (PMC13046165; doi:10.1371/journal.pbio.3003666)

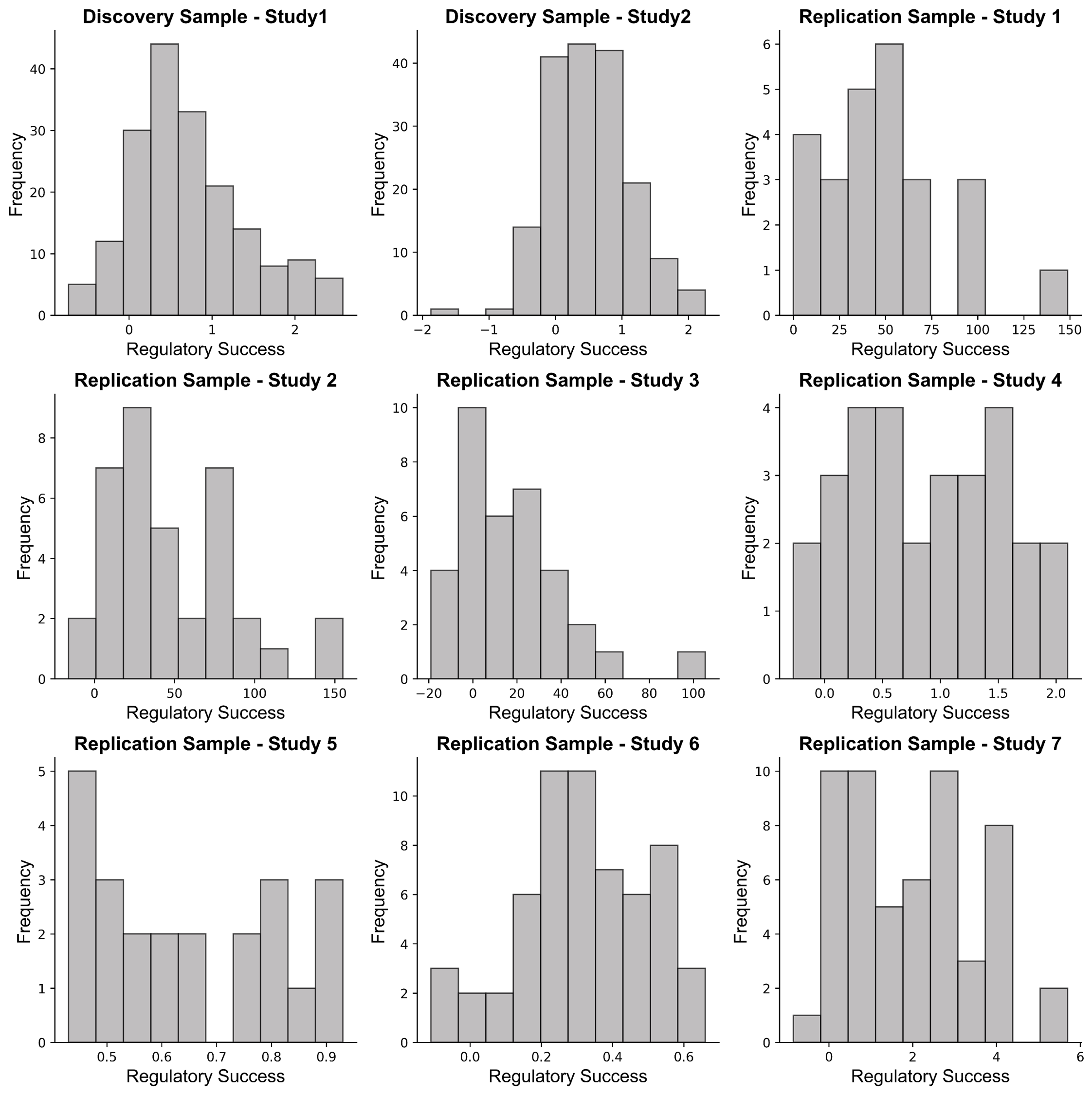

Supplement: S1 Fig — Higher positive values indicate more effective regulation of task-evoked emotional states (ratings in “Regulate” – “Look”). See S1 Table for an overview of the study-specific rating scales. Data underlying this figure can be found at: https://osf.io/yk85c/. (TIF) [file pbio.3003666.s001.tif]

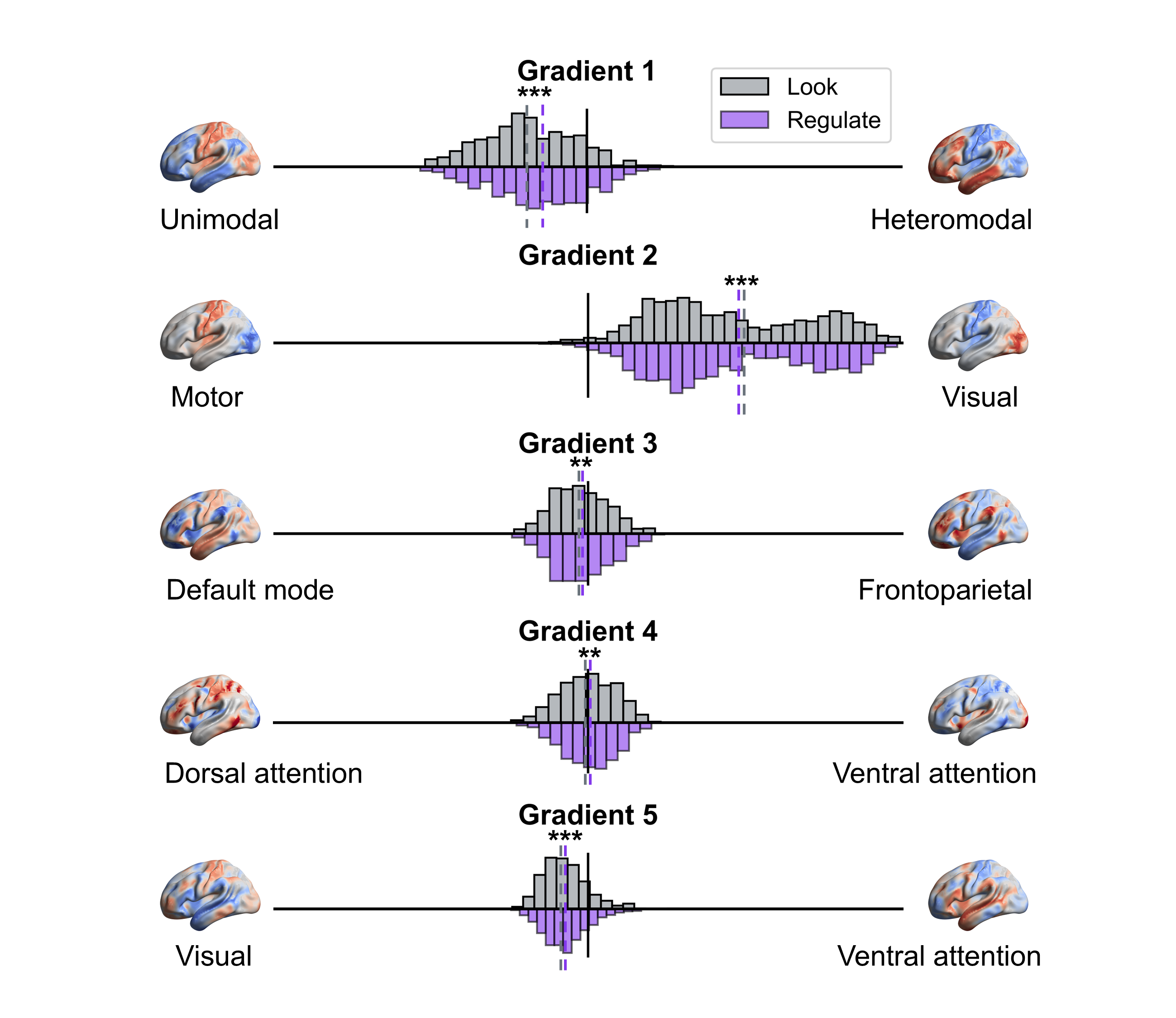

Supplement: S2 Fig — Dotted lines show condition-specific mean similarities with a principal gradient; asterisks indicate significant differences between both condition-specific gradient similarity scores along a particular gradient (Wilcoxon signed-rank tests, *p < 0.05; **p < 0.01; ***p < 0.001; Bonferroni-corrected). Data underlying this figure can be found at: https://osf.io/yk85c/. (TIF) [file pbio.3003666.s002.tif]

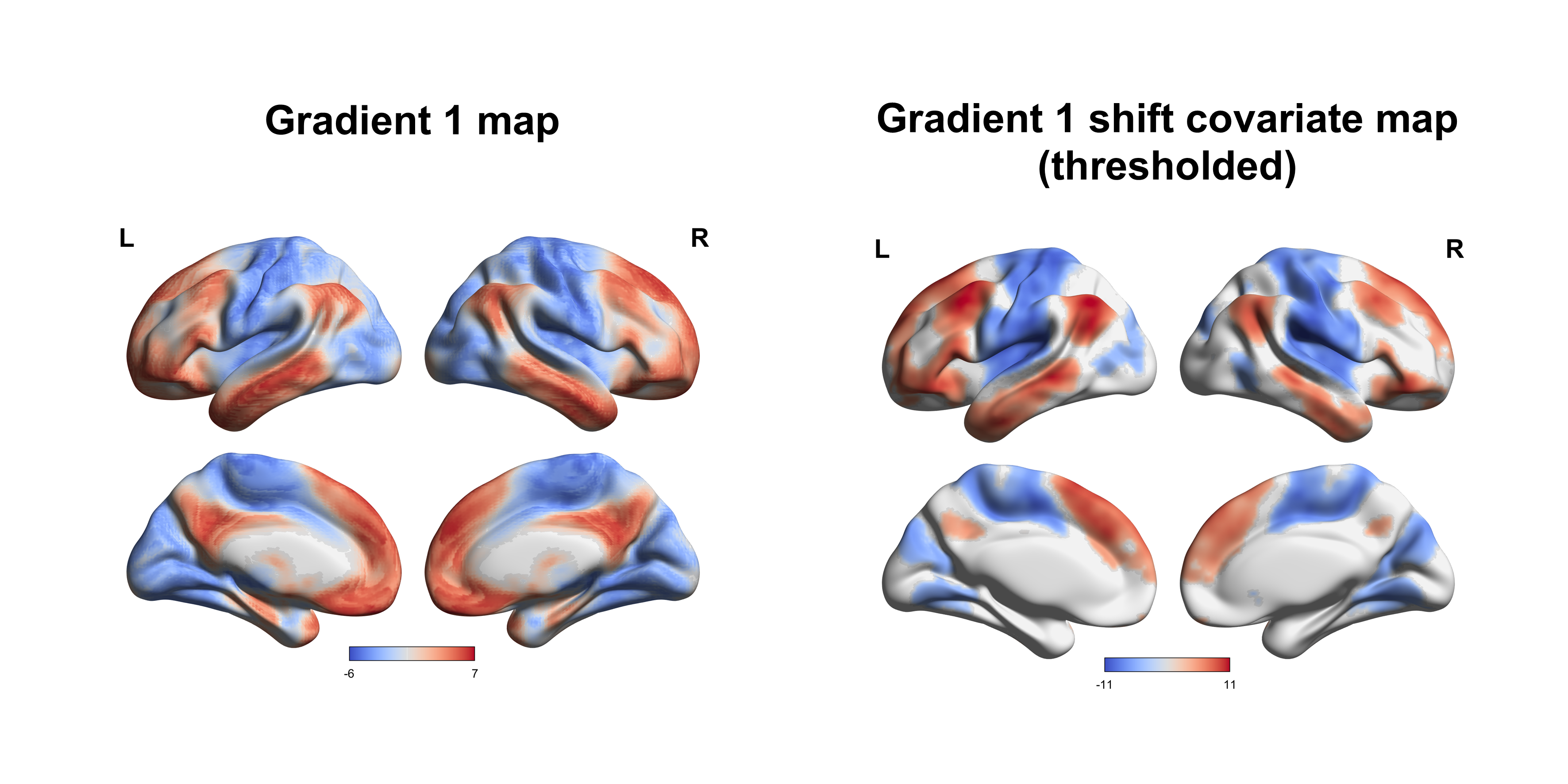

Supplement: S3 Fig — Left: Principal Gradient 1 map derived from independent resting-state fMRI data in the Human Connectome Project (HCP), depicting the unimodal–to–heteromodal cortical hierarchy for comparison. Right: Covariate t-map showing brain regions in which task-evoked activation during emotion regulation (Regulate > Look) covaries with participant-specific Gradient 1 shifts (ΔG1), thresholded at p < 0.001 (uncorrected) at the voxel level and cluster level FWE-corrected at p < 0.05. Warm (red) colors reflect positive associations; cool (blue) colors reflect negative associations. The results indicate that larger Gradient 1 shifts during emotion regulation are associated with greater activation in heteromodal regions and reduced activation in unimodal regions. Brain maps underlying this figure can be found at: https://osf.io/yk85c/. (TIF) [file pbio.3003666.s003.tif]

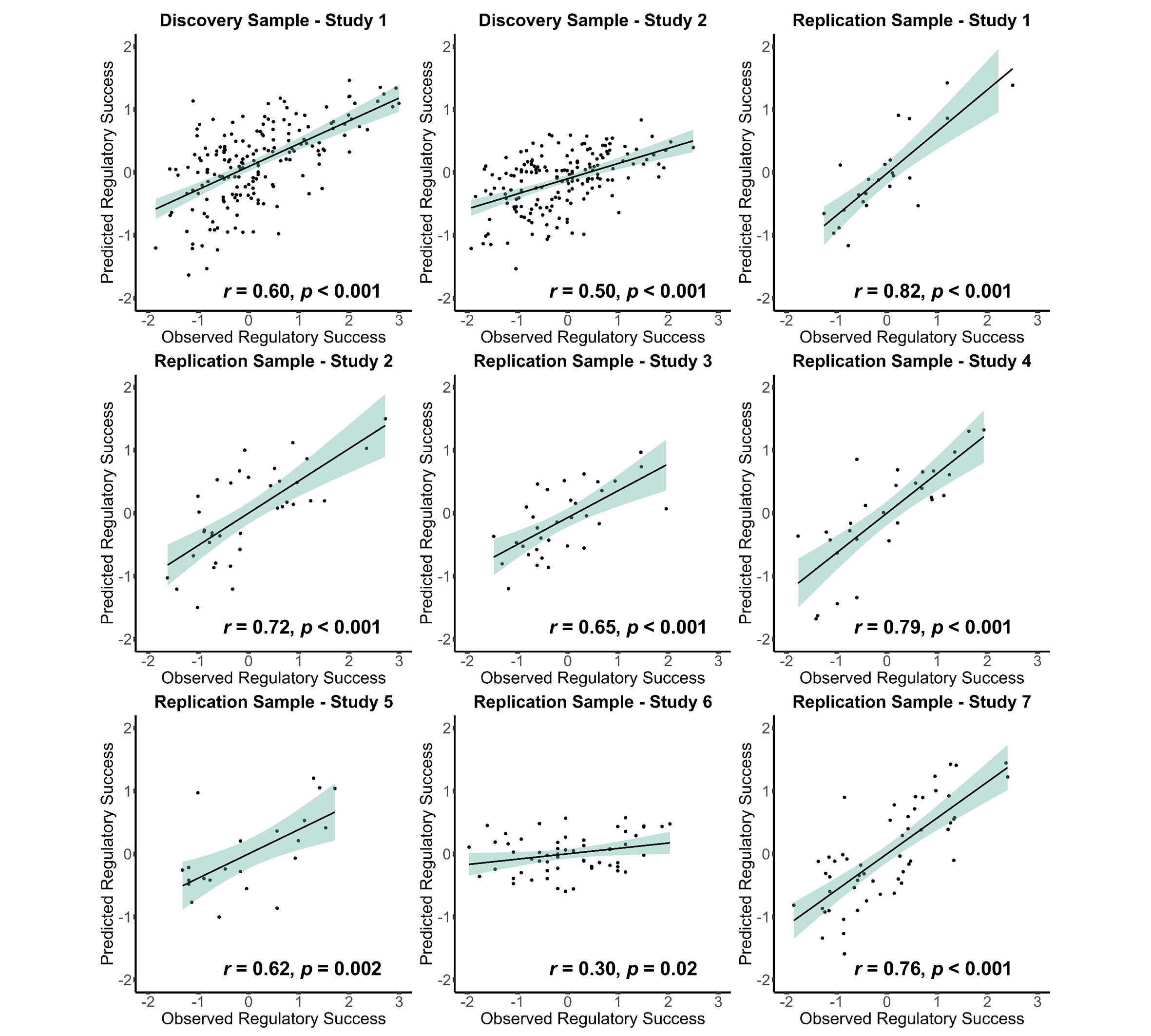

Supplement: S4 Fig — Data underlying this figure can be found at: https://osf.io/yk85c/. (TIF) [file pbio.3003666.s004.tif]

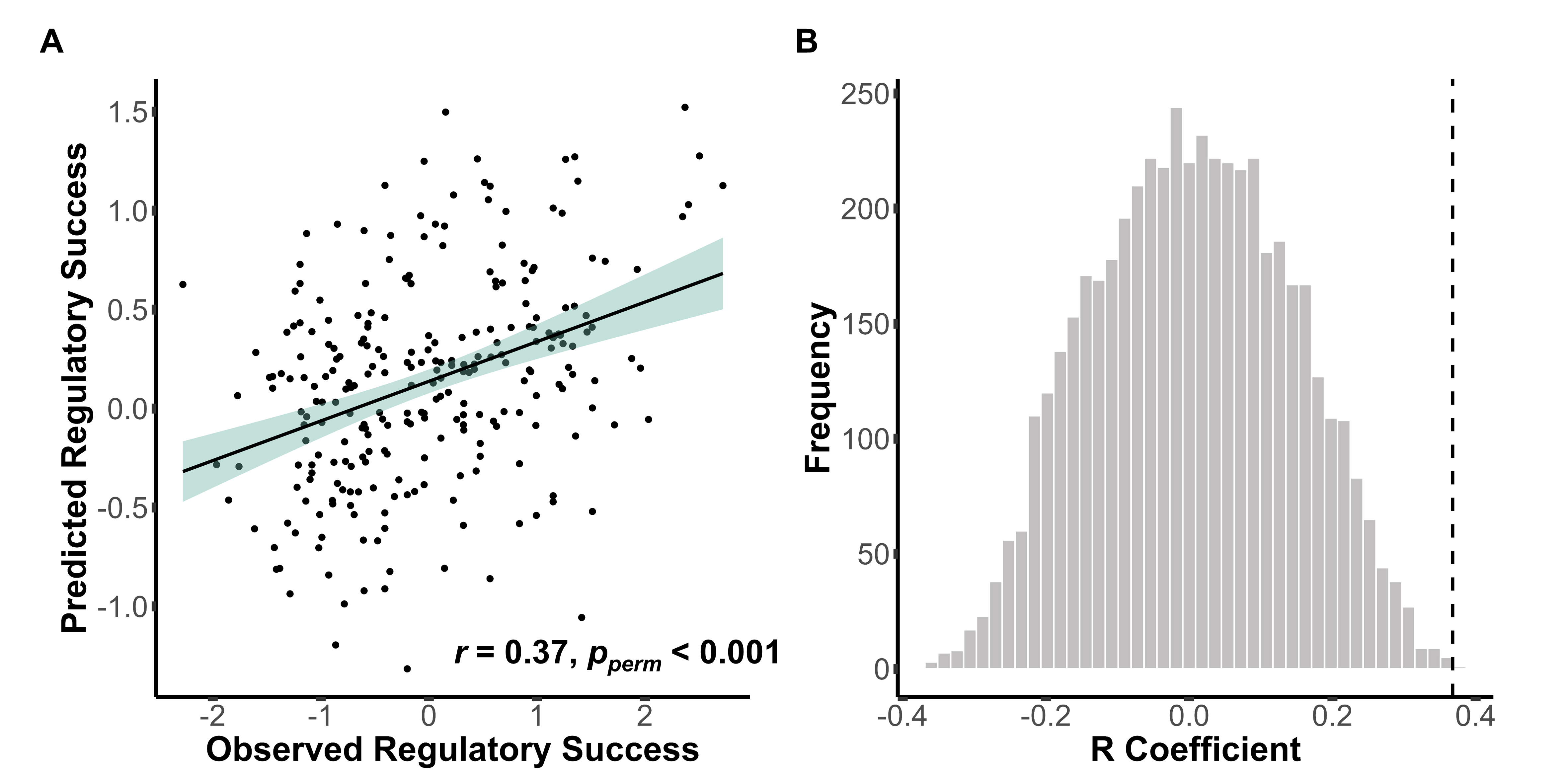

Supplement: S5 Fig — A. Pearson correlations between observed and predicted regulatory success scores using cross-sample prediction (r = 0.37, 95% CI [0.26, 0.47], pperm < 0.001), where we trained the predictive model on discovery sample data (DS) and applied the model on replication sample data (RS) to assess the out-of-sample generalizability. The statistical significance was assessed using a nonparametric permutation test. B. Visualization of the observed predictive accuracy of the out-of-sample prediction (dotted line) compared to the empirical null distribution of the prediction observed by chance (histogram). Data underlying this figure can be found at: https://osf.io/yk85c/. (TIF) [file pbio.3003666.s005.tif]

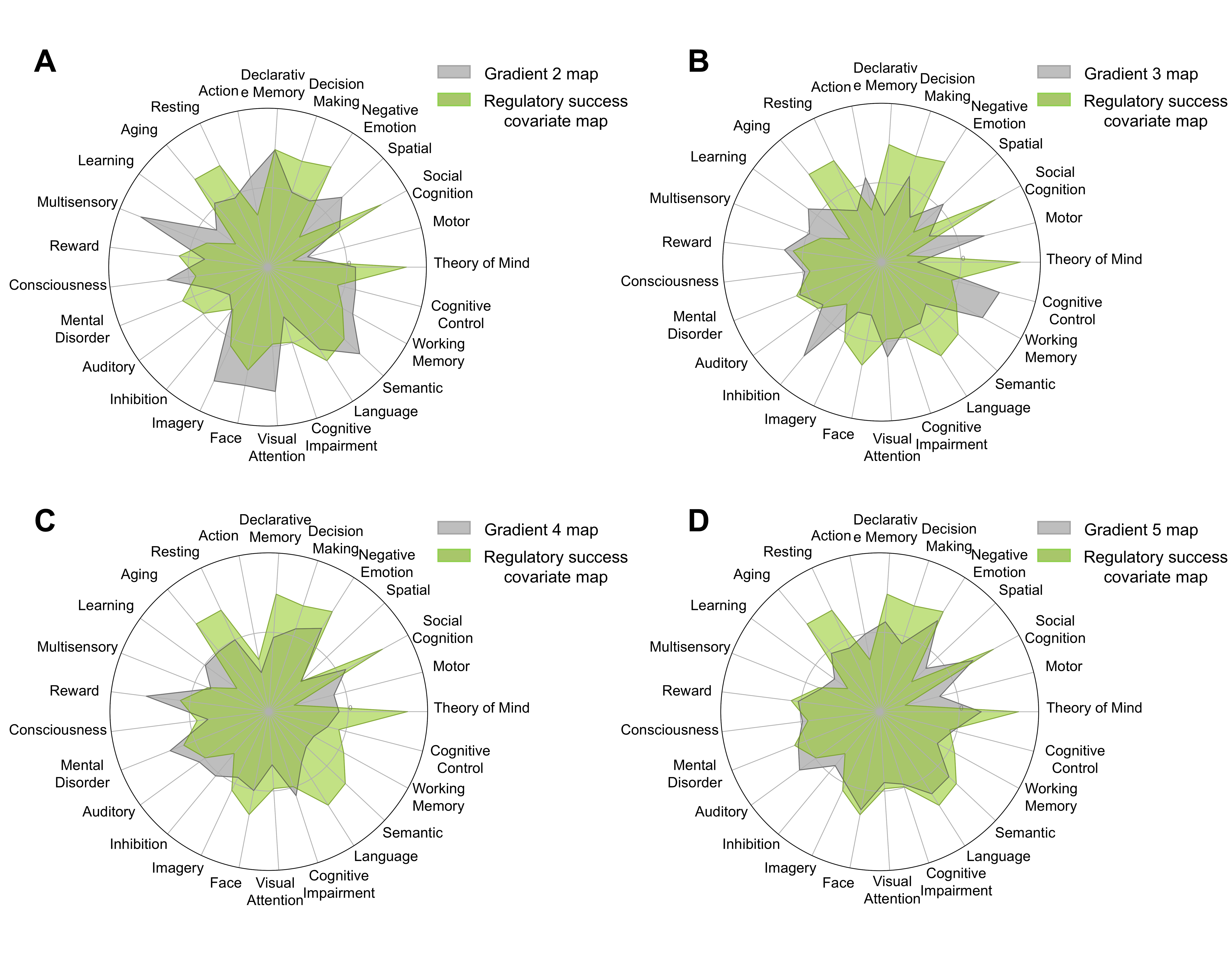

Supplement: S6 Fig — Data underlying this figure can be found in S6 Table. (TIF) [file pbio.3003666.s006.tif]

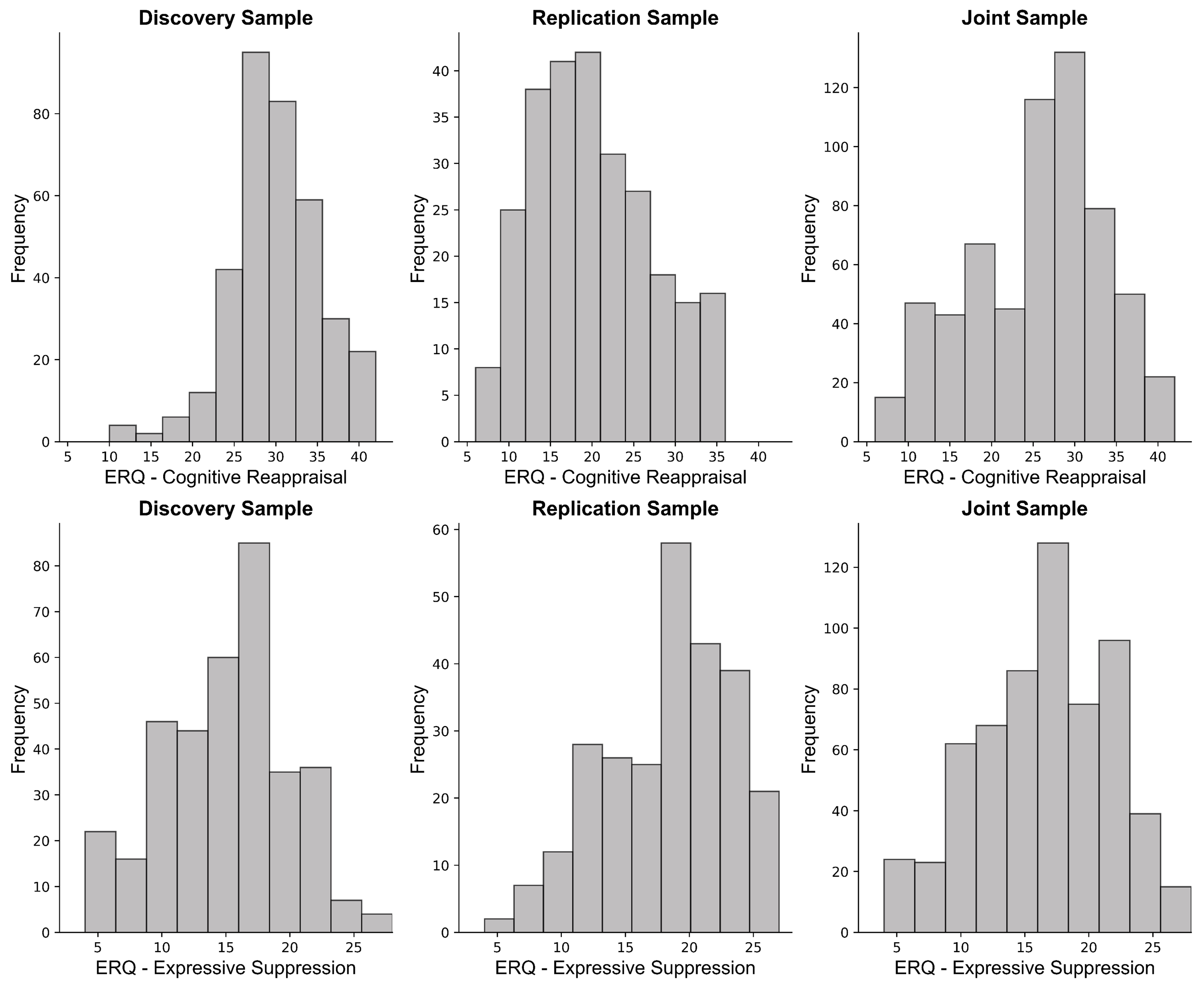

Supplement: S7 Fig — Mean ± SD [range]; ERQ-cognitive reappraisal: discovery sample: 29.86 ± 5.59; replication sample: 19.63 ± 7.25; joint sample: 25.56 ± 8.06; ERQ-expressive suppression: discovery sample: 14.87 ± 4.96; replication sample: 18.36 ± 4.90; joint sample: 16.35 ± 5.23. Data underlying this figure can be found at: https://osf.io/yk85c/. (TIF) [file pbio.3003666.s007.tif]

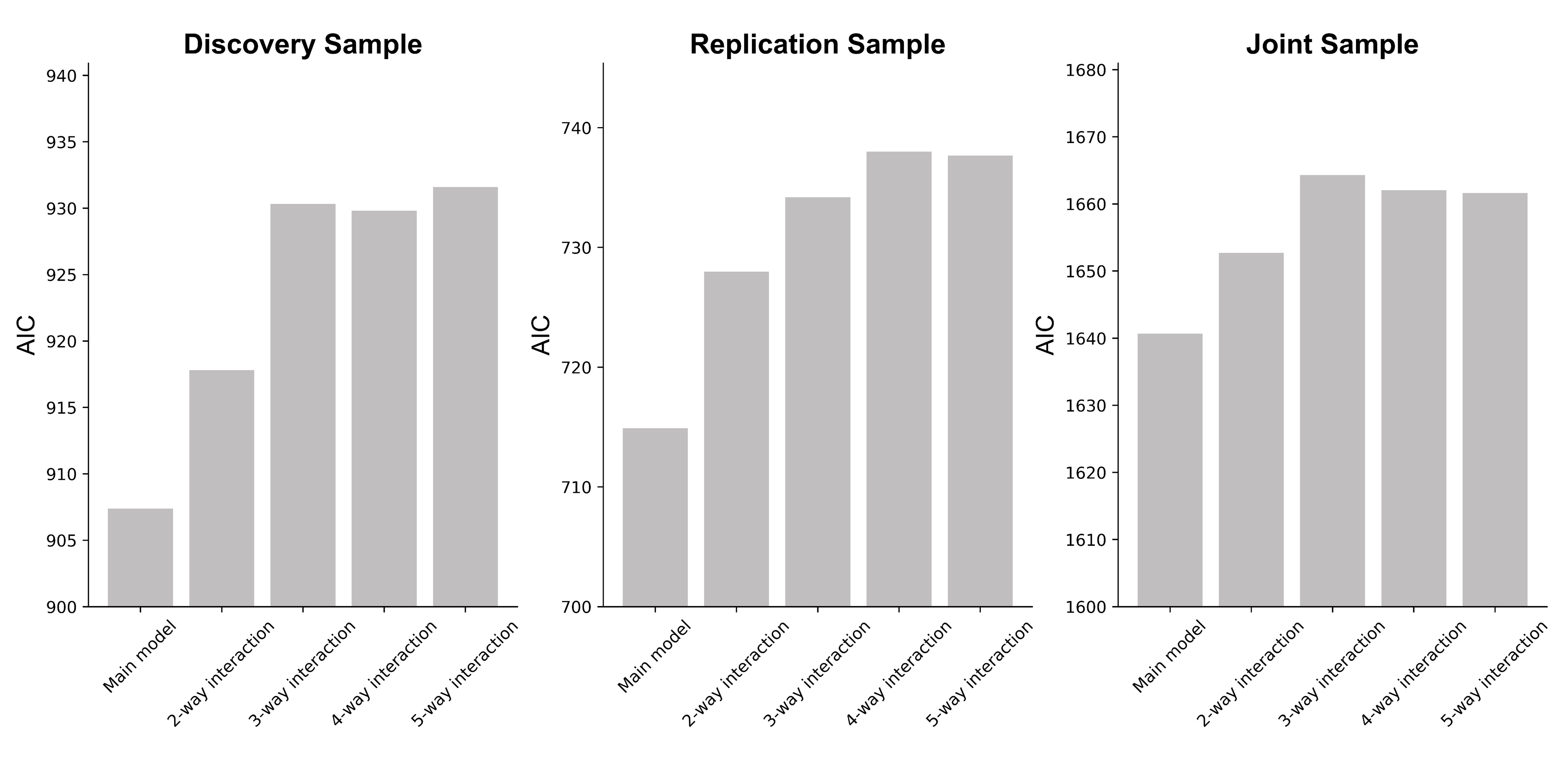

Supplement: S8 Fig — Across all samples, the main model (Eq. 1) showed the lowest AIC, indicating that it provided the best fit relative to models including interaction terms. Data underlying this figure can be found at: https://osf.io/yk85c/. (TIF) [file pbio.3003666.s008.tif]
