# Supplementary material for: Emotion regulation success involves systematic gradient-based reconfigurations of large-scale activation patterns in the human brain
Source: PLoS Biol. 2026 Apr 2;24(4):e3003666. doi: 10.1371/journal.pbio.3003666 (PMC13046165; doi:10.1371/journal.pbio.3003666)
Supplement: S1 Table — (DOCX) [file pbio.3003666.s009.docx]

## **S1 Table**. Study-specific emotional state ratings and regulatory success.

| Study | Rating Range | Ratings in “Look” | | Ratings in “Regulate” | Regulatory Success |
| --- | --- | --- | --- | --- | --- |
| Discovery Sample (DS) | | | | | |
| 1 | 1 to 5 | 2.51 ± 0.71  [1.15, 4.53] | 3.22 ± 0.64  [1.15, 4.73] | | 0.70 ± 0.68  [-0.73, 2.58] |
| 2 | 1 to 5 | 2.36 ± 0.65  [1.20, 4.57] | 2.86 ± 0.70  [1.0, 4.80] | | 0.51 ± 0.62  [-1.87, 2.25] |
| Replication Sample (RS) | | | | | |
| 1 | -200 to 200 | -72.09 ± 24.77  [-119.08, -15.46] | -22.58 ± 32.49  [-82.63, 34.83] | | 49.51 ± 34.65  [-0.04, 148.79] |
| 2 | -200 to 200 | -67.59 ± 39.97  [-134.44, 15.69] | -20.05 ± 36.06  [-95.28, 38.19] | | 47.54 ± 40.01  [-16.11, 155] |
| 3 | -200 to 200 | -18.74 ± 59.80  [-183.00, 108.21] | -1.38 ± 56.33  [-161.79, 106.79] | | 17.36 ± 25.02  [-19, 105.36] |
| 4 | -5 to 5 | -1.71 ± 0.86  [-3.29, 0.02] | -0.84 ± 0.65  [-2.4, 0.5] | | 0.86 ± 0.65  [-0.27, 2.1] |
| 5 | 1 to 4 | 2.14 ± 0.54  [1.21, 3.40] | 2.79 ± 0.54  [1.12, 3.46] | | 0.65 ± 0.17  [0.43, 0.93] |
| 6 | -5 to 5 | -1.12 ± 0.91  [-3.43, 1.83] | -0.82 ± 0.97  [-3.3, 1.43] | | 0.30 ± 0.18  [-0.11, 0.66] |
| 7 | -5 to 5 | -1.62 ± 0.94  [-3.7, 1.9] | 0.35 ± 1.21  [-2.18, 2.63] | | 1.97 ± 1.55  [-0.85, 5.7] |

*Note*: Higher positive values on the rating scale indicating more positive emotional states. Ratings and regulatory success scores reported as unstandardized mean ± standard deviations; the range of observed values [min, max] is reported in brackets.
