# Supplementary material for: Emotion regulation success involves systematic gradient-based reconfigurations of large-scale activation patterns in the human brain
Source: PLoS Biol. 2026 Apr 2;24(4):e3003666. doi: 10.1371/journal.pbio.3003666 (PMC13046165; doi:10.1371/journal.pbio.3003666)
Supplement: S2 Table — (DOCX) [file pbio.3003666.s010.docx]

## **S2 Table.** Dataset-specific statistical tests of projected activation maps against zero per principal gradient.

| Condition | Gradient 1 | Gradient 2 | Gradient 3 | Gradient 4 | Gradient 5 |
| --- | --- | --- | --- | --- | --- |
| *Discovery Sample (DS)* | |  |  |  |  |
| Look | p = 5.27 × 10^-39^  (6029) | p = 1.49 × 10^-58^  (109) | p = 1.04 × 10^-4^  (23036) | p = 1.47 × 10^-23^  (11879) | p = 3.27 × 10^-37^  (6640) |
| Regulate | p = 3.59 × 10^-20^  (13401) | p = 2.66 × 10^-58^  (178) | p = 1.42 × 10^-10^  (18583) | p = 7.84 × 10^-76^  (15548) | p = 1.41 × 10^-35^  (7210) |
| *Replication Sample (RS)* | |  |  |  |  |
| Look | p = 4.30 × 10^-44^  (28) | p = 3.14 × 10^-44^  (0) | p = 1.17 × 10^-38^  (1181) | p = 2.35 × 10^-26^  (4135) | p = 1.52 × 10^-34^  (2108) |
| Regulate | p = 2.90 × 10^-43^  (199) | p = 3.14 × 10^-44^  (0) | p = 5.50 × 10^-39^  (1109) | p = 1.59 × 10^-26^  (4090) | p = 1.58 × 10^-31^  (2824) |

*Note.* Bonferroni corrected p-values from dataset-specific non-parametric Wilcoxon signed-rank test against zero. Statistical V values are reported in brackets.
