# Supplementary material for: Emotion regulation success involves systematic gradient-based reconfigurations of large-scale activation patterns in the human brain
Source: PLoS Biol. 2026 Apr 2;24(4):e3003666. doi: 10.1371/journal.pbio.3003666 (PMC13046165; doi:10.1371/journal.pbio.3003666)
Supplement: S3 Table — (DOCX) [file pbio.3003666.s011.docx]

## **S3 Table.** Dataset-specific statistical tests of projected whole-brain activation maps in “Look” vs. “Regulate” per principal gradient.

| Condition | Gradient 1 | Gradient 2 | Gradient 3 | Gradient 4 | Gradient 5 |
| --- | --- | --- | --- | --- | --- |
| Discovery Sample (DS) | p = 2.09 × 10^-31^  (8839) | p = 1.06 × 10^-6^  (21569) | p = 3.55 × 10^-6^  (22004) | p = 5.49 × 10^-8^  (20572) | p = 0.48  (27926) |
| Replication Sample (RS) | p = 1.05 × 10^-10^  (9313) | p = 0.02  (13947) | p = 1  (17446) | p = 1  (16093) | p = 0.002  (13164) |

*Note.* Bonferroni corrected p-values from dataset-specific non-parametric Wilcoxon signed-rank test against zero. Statistical V values are reported in brackets.
