# Supplementary material for: Emotion regulation success involves systematic gradient-based reconfigurations of large-scale activation patterns in the human brain
Source: PLoS Biol. 2026 Apr 2;24(4):e3003666. doi: 10.1371/journal.pbio.3003666 (PMC13046165; doi:10.1371/journal.pbio.3003666)
Supplement: S4 Table — (DOCX) [file pbio.3003666.s012.docx]

## **S4 Table.** Significant clusters showing covariation between Gradient-1 Shifts (ΔG1) and task-evoked activation (Regulate > Look).

| Brain region | Side | k | t | MNI | | |
| --- | --- | --- | --- | --- | --- | --- |
|  |  |  |  | x | y | z |
| *Positive association* |  |  |  |  |  |  |
| Dorsal medial prefrontal cortex (dmPFC), extending to the ventral lateral prefrontal cortex (vlPFC), and dorsal lateral prefrontal cortex (dlPFC) | L & R | 5463 | 8.91 | -12 | 40 | 48 |
| Temporal parietal junction (TPJ) | L | 1043 | 9.01 | -48 | -66 | 40 |
| TPJ | R | 380 | 7.53 | 54 | -62 | 34 |
| Inferior frontal gyrus (IFG) | L | 554 | 7.49 | -46 | 26 | -10 |
| Caudate | L | 91 | 6.21 | -12 | 12 | 10 |
| Caudate | R | 58 | 5.47 | 12 | 8 | 16 |
| Middle temporal gyrus (MTG) | L | 114 | 6.53 | -54 | -36 | 6 |
| Precuneus | L & R | 359 | 5.82 | 2 | -60 | 34 |
| Middle frontal gyrus (MFG) | L | 19 | 5.11 | -32 | 54 | 6 |
| *Negative association* |  |  |  |  |  |  |
| Supplementary motor cortex, extending to the postcentral gyrus, middle cingulate cortex, central operculum, and insula | L & R | 15058 | 9.28 | 42 | 2 | 10 |
| Cuneus | R | 2210 | 7.77 | 14 | -76 | 24 |
| Lingual gyrus | L | 167 | 5.35 | -12 | -56 | -6 |

*Note.* Results are reported at p < 0.001 (uncorrected) at the voxel level and FWE-corrected at p < 0.05 at the cluster-level; only peak activations of clusters are reported; L left hemisphere, R right hemisphere, k cluster size in voxels, MNI Montreal Neurological Institute.
