# Supplementary material for: Emotion regulation success involves systematic gradient-based reconfigurations of large-scale activation patterns in the human brain
Source: PLoS Biol. 2026 Apr 2;24(4):e3003666. doi: 10.1371/journal.pbio.3003666 (PMC13046165; doi:10.1371/journal.pbio.3003666)
Supplement: S5 Table — (DOCX) [file pbio.3003666.s013.docx]

## **S5 Table.** Significant clusters showing covariation between participants’ regulatory success and task-evoked activation (Regulate > Look).

| Brain region | Side | k | t | MNI | | |
| --- | --- | --- | --- | --- | --- | --- |
|  |  |  |  | x | y | z |
| *Positive association* |  |  |  |  |  |  |
| Inferior frontal gyrus (IFG) † | L | 227 | 3.70 | -38 | 14 | 24 |
| IFG | L | 138 | 3.80 | -52 | 22 | 10 |
| IFG | L | 33 | 3.58 | -36 | 30 | -8 |
| IFG | R | 27 | 3.57 | 58 | 22 | 12 |
| Middle temporal gyrus (MTG) | L | 113 | 4.24 | -50 | -36 | -4 |
| MTG | R | 13 | 3.41 | 58 | -36 | -2 |
| MTG | R | 10 | 3.23 | 58 | -12 | -16 |
| Superior temporal sulcus (STS) | L | 67 | 4.14 | -58 | -64 | 20 |
| STS | R | 24 | 3.80 | 42 | -38 | 4 |
| STS | R | 117 | 3.63 | 58 | -56 | 28 |
| Superior frontal gyrus (SFG) | R | 39 | 3.73 | 12 | 28 | 52 |
| SFG | R | 29 | 3.70 | 18 | 42 | 50 |
| SFG | L | 19 | 3.59 | -14 | 32 | 42 |
| SFG | L | 48 | 3.46 | -16 | 56 | 26 |
| Temporal pole (TP) | R | 26 | 3.66 | 2 | 20 | -20 |
| Ventral medial prefrontal cortex (vmPFC) | L | 23 | 3.41 | -10 | 46 | -10 |
| *Negative association* |  |  |  |  |  |  |
| Insula † | R | 307 | 4.32 | 44 | -24 | 24 |
| Insula | R | 10 | 3.29 | 38 | -12 | -6 |
| Supplementary motor area (SMA) | L | 153 | 4.80 | -10 | -10 | 58 |
| SMA | R | 11 | 3.31 | 10 | 6 | 44 |
| Supramarginal gyrus (SMG) | L | 180 | 4.26 | -60 | -26 | 28 |
| Posterior cingulate cortex (PCC) | L | 82 | 4.31 | -16 | -34 | 38 |
| PCC | R | 32 | 3.68 | 14 | -30 | 42 |
| Postcentral gyrus | L | 51 | 4.15 | -20 | -38 | 62 |
| Postcentral gyrus | L | 19 | 3.40 | -48 | -26 | 56 |
| Central operculum | R | 91 | 4.07 | 50 | 4 | 8 |
| Central operculum | L | 87 | 3.72 | -50 | 0 | 8 |
| Premotor cortex | R | 16 | 3.62 | 56 | 6 | 32 |
| Caudate | R | 17 | 3.51 | 22 | 18 | 20 |
| Parietal operculum | L | 13 | 3.22 | -46 | -26 | 18 |

*Note.* Results are reported at a statistical threshold of *p* < 0.001 uncorrected, with a cluster threshold of 10 voxels; † indicates results significant at *p* < 0.05, FWE corrected at the cluster level; only peak activations of clusters are reported; L left hemisphere, R right hemisphere, k cluster size in voxels, MNI Montreal Neurological Institute.
