# Supplementary material for: Emotion regulation success involves systematic gradient-based reconfigurations of large-scale activation patterns in the human brain
Source: PLoS Biol. 2026 Apr 2;24(4):e3003666. doi: 10.1371/journal.pbio.3003666 (PMC13046165; doi:10.1371/journal.pbio.3003666)
Supplement: S6 Table — (DOCX) [file pbio.3003666.s014.docx]

## **S6 Table.** Neurosynth meta-analytical decoding results.

| Terms | Gradient 1 | Gradient 2 | Gradient 3 | Gradient 4 | Gradient 5 | RS covariate |
| --- | --- | --- | --- | --- | --- | --- |
| Declarative Memory | 0.25 | 0.24 | -0.21 | -0.03 | 0.07 | 0.14 |
| Decision Making | 0.25 | -0.01 | 0.07 | 0.05 | -0.05 | 0.18 |
| Negative Emotion | 0.27 | -0.01 | -0.17 | 0.12 | 0.18 | 0.17 |
| Spatial | -0.30 | 0.14 | 0.03 | -0.22 | -0.10 | -0.30 |
| Social Cognition | 0.35 | 0.02 | -0.16 | 0.05 | 0.17 | 0.32 |
| Motor | -0.36 | -0.24 | 0.17 | -0.08 | -0.11 | -0.20 |
| Theory of Mind | 0.39 | 0.05 | -0.27 | -0.06 | 0.14 | 0.36 |
| Cognitive Control | -0.04 | 0.07 | 0.27 | -0.12 | -0.02 | 0.17 |
| Working Memory | 0.04 | 0.11 | 0.23 | -0.18 | -0.09 | 0.21 |
| Semantic | 0.04 | 0.30 | -0.12 | -0.18 | 0.10 | 0.04 |
| Language | 0.05 | 0.11 | -0.04 | -0.12 | 0.11 | 0.25 |
| Cognitive Impairment | 0.06 | -0.17 | -0.05 | 0.06 | -0.02 | 0.01 |
| Visual Attention | -0.06 | 0.28 | 0.10 | -0.16 | -0.05 | 0.04 |
| Face | 0.07 | 0.26 | -0.16 | 0.01 | 0.13 | -0.02 |
| Imagery | 0.08 | 0.29 | -0.15 | -0.04 | 0.00 | -0.06 |
| Inhibition | -0.12 | -0.15 | 0.27 | 0.03 | -0.06 | 0.12 |
| Auditory | -0.13 | -0.21 | -0.04 | 0.04 | 0.12 | 0.06 |
| Mental Disorder | 0.14 | -0.13 | 0.05 | 0.17 | 0.05 | 0.05 |
| Consciousness | -0.15 | 0.14 | -0.01 | -0.11 | -0.01 | -0.11 |
| Reward | 0.16 | -0.10 | 0.12 | 0.28 | 0.02 | 0.04 |
| Multisensory | -0.21 | 0.36 | -0.01 | -0.11 | -0.10 | -0.30 |
| Learning | -0.21 | -0.10 | 0.07 | -0.01 | -0.15 | -0.26 |
| Aging | 0.23 | 0.02 | -0.07 | -0.01 | -0.02 | 0.19 |
| Resting | 0.24 | -0.02 | -0.14 | 0.00 | -0.06 | 0.19 |
| Action | -0.24 | 0.07 | 0.04 | -0.25 | 0.00 | -0.13 |

*Note.* *RS*, regulatory success. The RS covariate map was estimated using SPM12 (<https://www.fil.ion.ucl.ac.uk/spm/>) at the group level (using participant-specific RS scores as a covariate in a simple *t*-test as implemented in SPM12; unthresholded whole-brain *t*-maps).
