# Supplementary material for: Emotion regulation success involves systematic gradient-based reconfigurations of large-scale activation patterns in the human brain
Source: PLoS Biol. 2026 Apr 2;24(4):e3003666. doi: 10.1371/journal.pbio.3003666 (PMC13046165; doi:10.1371/journal.pbio.3003666)
Supplement: S7 Table — (DOCX) [file pbio.3003666.s015.docx]

## **S7 Table.** Regulation-related shifts of activation patterns along Gradient 1 in the laboratory emotion regulation task predict average negative affect in daily life.

| Predictor | Estimate *(SE)* | *t* | *p* | *95% CI* |
| --- | --- | --- | --- | --- |
| Gradient 1 shift (ΔG1) | 1.19 (0.38) | 3.13 | 0.002 | [0.44, 1.94] |
| Gradient 2 shift (ΔG2) | -0.94 (0.82) | -1.15 | 0.25 | [-2.56, 0.68] |
| Gradient 3 shift (ΔG3) | 1.04 (0.74) | 1.41 | 0.16 | [-0.42, 2.51] |
| Gradient 4 shift (ΔG4) | 1.21 (0.73) | 1.64 | 0.10 | [-0.25, 2.66] |
| Gradient 5 shift (ΔG5) | 0.62 (0.69) | 0.91 | 0.36 | [-0.73, 1.98] |
| Emotional reactivity | -0.04 (0.03) | -1.07 | 0.29 | [-0.11, 0.03] |
| Age | -0.01 (0.01) | -1.49 | 0.13 | [-0.02, 0.003] |
| Sex | 0.10 (0.05) | 1.93 | 0.06 | [-0.002, 0.21] |

*Note.* Average negative affect in daily life was assessed based on self-reports assessed in momentary ecological assessments on participants phones over a 1-week period in *n* = 55 of the RS sample (Wang et al., 2025).

**References:**

1. Wang, R., et al., *Patterns of Ongoing Thought Shape Emotion Regulation and Well-Being in Daily Life.* PsyArXiv, 2025.
