# Supplementary material for: Emotion regulation success involves systematic gradient-based reconfigurations of large-scale activation patterns in the human brain
Source: PLoS Biol. 2026 Apr 2;24(4):e3003666. doi: 10.1371/journal.pbio.3003666 (PMC13046165; doi:10.1371/journal.pbio.3003666)
Supplement: S8 Table — (DOCX) [file pbio.3003666.s016.docx]

## **S8 Table.** AIC values of model comparisons (models with interaction terms).

| Model | Discovery Sample | Replication Sample | Joint Sample |
| --- | --- | --- | --- |
| Main model | 907.38 | 714.90 | 1640.69 |
| Two-way interactions | 917.80 | 727.98 | 1652.70 |
| Three-way interactions | 930.33 | 734.19 | 1664.30 |
| Four-way interactions | 929.82 | 737.99 | 1662.04 |
| Five-way interactions | 931.59 | 737.66 | 1661.60 |

*Note*. Smaller AIC (Akaike Information Criterion) values indicate better model fit.
