# Supplementary material for: Emotion regulation success involves systematic gradient-based reconfigurations of large-scale activation patterns in the human brain
Source: PLoS Biol. 2026 Apr 2;24(4):e3003666. doi: 10.1371/journal.pbio.3003666 (PMC13046165; doi:10.1371/journal.pbio.3003666)
Supplement: S9 Table — (DOCX) [file pbio.3003666.s017.docx]

# **S9 Table.** AIC values of the model comparisons (models with ED, Euclidean distance).

| Model | Discovery Sample | Replication Sample | Joint Sample |
| --- | --- | --- | --- |
| Main model | 907.38 | 714.90 | 1640.69 |
| 5-D ED model | 911.36 | 718.05 | 1663.04 |
| 3-D ED model | 910.79 | 721.15 | 1665.01 |

*Note*. Smaller AIC (Akaike Information Criterion) values indicate better model fit.
