# Supplementary material for: Emotion regulation success involves systematic gradient-based reconfigurations of large-scale activation patterns in the human brain
Source: PLoS Biol. 2026 Apr 2;24(4):e3003666. doi: 10.1371/journal.pbio.3003666 (PMC13046165; doi:10.1371/journal.pbio.3003666)
Supplement: S10 Table — (DOCX) [file pbio.3003666.s018.docx]

## **S10 Table.** Overview of the study-specific MRI acquisition parameters and preprocessing of fMRI data.

| **Study** | **Scanner** | **Ethics (Approval number)** | **EPI sequence** | **Processing details** |  |
| --- | --- | --- | --- | --- | --- |
| Discover  Sample |  |  |  |  |  |
| 1 | Siemens 3T Trio | IRB of The University of Pittsburgh (07040037) | TR = 2000 ms, TE = 28 ms, matrix size 64 * 64, voxel size 3 * 3 * 3 mm, flip angle = 90°, filed of view = 205 * 205 mm | SPM 12; slice-time correction, realignment to the first image, coregistration to the respective structural image of the participant, spatial normalization to the standard EPI template (2*2*2 mm voxels, Montreal Neurological Institute, MNI), and smoothing with an isotropic Gaussian kernel (6 mm full-width at half-maximum). |  |
| 2 | Siemens 3T Trio | IRB of The University of Pittsburgh (07110287) | TR = 2000 ms, TE = 28 ms, matrix size 64 * 64, voxel size 3 * 3 * 3 mm, flip angle = 90°, filed of view = 205 * 205 mm | SPM 12; slice-time correction, realignment to the first image, coregistration to the respective structural image of the participant, spatial normalization to the standard EPI template (2*2*2 mm voxels, Montreal Neurological Institute, MNI), and smoothing with an isotropic Gaussian kernel (6 mm full-width at half-maximum). |  |
| Replication  Sample | |  |  |  |  |
| 1 | Siemens 7T | Medical University of Vienna (1309/2018) | TR = 1400 ms, TE = 23 ms, voxel size 1.5 * 1.5 * 1.0 mm, 78 slices, flip angle = 62°, filed of view = 192 * 192 * 97.5 mm | SPM12; slice-time correction, realignment, coregistration to the respective structural image of the participant, spatial normalization to the standard EPI template (Montreal Neurological Institute, MNI), and smoothing with an isotropic Gaussian kernel (6 mm full-width at half-maximum). |  |
| 2 | Siemens 3T Trio | Freie Universität Berlin (NA) | TR = 2000 ms, TE = 30 ms, matrix size 64 * 64, voxel size 3 * 3 * 3 mm, 37 slices, flip angle = 90°, filed of view = 192 * 192 mm | SPM12; slice time correction was included during the preprocessing of the fMRI data. In addition, standard preprocessing involved realignment to the mean image of the first run, spatial normalization to the standard EPI template (MNI template), and spatial smoothing with an 8 mm full-width at half-maximum (FWHM) isotopic Gaussian kernel. |  |
| 3 | Siemens 3T Trio | Freie Universität Berlin (NA) | TR = 2000 ms, TE = 30 ms, matrix size 64 * 64, voxel size 3 * 3 * 3 mm, 37 slices, flip angle = 90°, filed of view = 192 * 192 mm | SPM12; slice time correction was included during the preprocessing of the fMRI data. In addition, standard preprocessing involved realignment to the mean image of the first run, spatial normalization to the standard EPI template (MNI template), and spatial smoothing with an 8 mm full-width at half-maximum (FWHM) isotopic Gaussian kernel. |  |
| 4 | Siemens 3T Trio | Freie Universität Berlin (NA) | TR = 2000 ms, TE = 30 ms, matrix size 64 * 64, voxel size 3 * 3 * 3 mm, 37 slices, flip angle = 90°, filed of view = 192 * 192 mm | SPM12; slice time correction was included during the preprocessing of the fMRI data. In addition, standard preprocessing involved realignment to the mean image of the first run, spatial normalization to the standard EPI template (MNI template), and spatial smoothing with an 8 mm full-width at half-maximum (FWHM) isotopic Gaussian kernel. |  |
| 5 | Siemens 3T Trio | German Psychological Society (NA) | TR = 2000 ms, TE = 30 ms, matrix size 64 * 64, voxel size 3 * 3 * 3 mm, 37 slices, flip angle = 90°, filed of view = 192 * 192 mm | SPM8; slice time correction, realignment to the mean image, coregistration to the individual T1-weighted anatomical images as well as spatial normalization to the standard EPI template, reslicing to 3*3*3 mm voxels (Montreal Neurological Institute, MNI template, as implemented in SPM8). Spatial smoothing was performed using an 8mm full-width at half-maximum (FWHM) isotopic Gaussian kernel. |  |
| 6 | Siemens 3T Trio | German Psychological Society (NA) | TR = 2000 ms, TE = 30 ms, matrix size 64 * 64, voxel size 3 * 3 * 3 mm, 37 slices, flip angle = 90°, filed of view = 192 * 192 mm | SPM8; slice time correction, realignment to the mean image, coregistration to the individual T1-weighted anatomical images as well as spatial normalization to the standard EPI template, reslicing to 3*3*3 mm voxels (Montreal Neurological Institute, MNI template, as implemented in SPM8). Spatial smoothing was performed using an 8mm full-width at half-maximum (FWHM) isotopic Gaussian kernel. |  |
| 7 | Siemens 3T Prisma | HSREB of Queen’s University (PSYC-267-22) | TR = 1500 ms, TE = 30 ms, matrix size 84 * 84, voxel size 2.5 * 2.5 * 2.5 mm, flip angle = 65°, filed of view = 210 * 210 mm | SPM 12; slice-time correction, realignment to the first image, coregistration to the respective structural image of the participant, spatial normalization to the standard EPI template (2*2*2 mm voxel, Montreal Neurological Institute, MNI), and smoothing with an isotropic Gaussian kernel (6 mm full-width at half-maximum). |  |
