# Supplementary material for: Emotion regulation success involves systematic gradient-based reconfigurations of large-scale activation patterns in the human brain
Source: PLoS Biol. 2026 Apr 2;24(4):e3003666. doi: 10.1371/journal.pbio.3003666 (PMC13046165; doi:10.1371/journal.pbio.3003666)
Supplement: Attachment — Submitted filename: response_letter_4submission.docx [file pbio.3003666.s021.docx]

**Response Letter**

Dear Editors and Reviewers,

We sincerely appreciate your thoughtful and insightful comments on our manuscript. In response, we have substantially revised both the main text and the supplemental materials, and we believe these revisions have significantly strengthened the work.

Following the reviewer’s constructive feedback, we removed causal language, improved the clarity of presentation throughout the manuscript, and added relevant references. We also implemented several supplemental analyses, examining (i) the association between emotion regulation-related neural activation and shifts along Gradient 1, and (ii) the relationship between *region-specific* gradient shifts and individual differences in regulatory success.

We are very grateful to the reviewers for their time and effort in providing such careful and constructive evaluations. We hope that our revisions satisfactorily address all comments and concerns.

Below, you will find our detailed point-by-point responses to each comment. The original comments of the review team are presented in *italics,* and we quote the relevant text of the revised manuscript where appropriate.

Sincerely,
Anita Tusche, Carmen Morawetz, Remi Janet, and Ruien Wang

**EDITOR COMMENTS**

*Dear Mr. Wang,*

*Thank you for your patience while your manuscript "Emotion Regulation in the Gradient Framework: Large-Scale Brain Organization Shapes Individual Differences in Reappraisal Success" was peer-reviewed at PLOS Biology. It has now been evaluated by the PLOS Biology editors, an Academic Editor with relevant expertise, and by several independent reviewers.*

*In light of the reviews, which you will find at the end of this email, we would like to invite you to revise the work to thoroughly address the reviewers' reports.*

*As you will see below, the reviewers think that the study is overall well executed and provides important insights, but they also raise concerns about the clarity of the presentation and the (potentially) exploratory type of the analysis and that the claims regarding the involvement of the entire brain are currently not sufficiently supported. We therefore ask you to address the reviewer concerns by improving clarity of the presentation, transparency of the analysis, and add the analyses as suggested by Reviewer 3.*

*Given the extent of revision needed, we cannot make a decision about publication until we have seen the revised manuscript and your response to the reviewers' comments. Your revised manuscript is likely to be sent for further evaluation by all or a subset of the reviewers.*

*In addition to these revisions, you will need to complete some formatting changes, which you will receive in a follow up email. A member of our team will be in touch with a set of requests shortly.*

*We expect to receive your revised manuscript within 3 months. Please email us (plosbiology@plos.org) if you have any questions or concerns, or would like to request an extension.*

*At this stage, your manuscript remains formally under active consideration at our journal; please notify us by email if you do not intend to submit a revision so that we may withdraw it.*

*Thank you again for your submission to our journal. We hope that our editorial process has been constructive thus far, and we welcome your feedback at any time. Please don't hesitate to contact us if you have any questions or comments.*

*Sincerely,*

*Christian*

*Christian Schnell, PhD
Senior Editor
PLOS Biology*
[*cschnell@plos.org*](mailto:cschnell@plos.org)

**Response:**

We thank the editor for the positive assessment of our work and for the invitation to resubmit. As a response, we have now carefully ensured the use of non-causal language throughout the revised manuscript. For example, we have replaced “shapes” with terms such as “is associated with,” “covaries with,” “relates to,” “corresponds to,” and “reflects,” and made a minor modification to the title to remove the word “shape”. We have also clarified our a priori hypothesis in the revised manuscript, added several methodological clarifications throughout the paper (including modifications to the main text, figures and tables), and incorporated additional relevant references on principal gradients in the brain in the introduction.

Regarding the concern that whole-brain involvement in the gradient approach is not sufficiently supported, we have rerun the analysis using a *region-wise* analysis approach suggested by Reviewer 3. We applied cortical (Schaefer 400 atlas, 400 regions) and subcortical (Harvard-Oxford Subcortical Atlas, 14 regions) parcellation to derive region-specific gradient shifts and examine their associations with variance in emotion regulation success. After controlling for multiple comparisons, no cortical or subcortical region shows a significant association with regulatory success. This supplemental result supports our main conclusion that the observed effects are not driven by isolated brain regions but instead reflect a systematic, large-scale neural reconfiguration along principal gradients that tracks individual differences in reappraisal success.

Further details on all revisions and supplemental analyses are provided below in our point-by-point responses to the reviewers. Overall, we believe our current approach provides a robust and validated framework for understanding emotion regulation and individual differences in emotion regulation success from a large-scale brain organization perspective.

Finally, we have also completed the formatting changes for the resubmission, as requested by the editorial team.

**REVIEWER COMMENTS**

**REVIEWER 1**

*In this work, the authors applied spatial gradient analysis to brain activation during emotion regulation and found that shifts along a gradient differentiating unimodal and heteromodal brain areas can predict reappraisal success. This provides a novel angle for understanding emotion regulation success within a network framework. The study is well-powered, and the out-of-sample prediction performance is impressive. The writing is clear, and the analyses are clean. I have a few suggestions.*

**Response:** We sincerely thank the reviewer for the overall positive assessment of our work! We have addressed each point in detail below and believe that the corresponding changes have significantly strengthened the revised manuscript.

*The interpretation that large-scale brain organization "shapes" individual differences in reappraisal success seems to imply causality, whereas the analysis is correlational.*

**Response:** We thank the reviewer for raising this important point. We agree that the original phrasing using “shapes” implied directionality and a causal relationship that is not supported by our analysis. To address this, we have revised the manuscript throughout, replacing “shapes” with non-causal terms such as “is associated with,” “covaries with,” “relates to,” “corresponds to,” and “reflects,” to more accurately describe the relationship between large-scale brain organization and individual differences in regulatory success. We have also updated the manuscript title, removing “shapes” and replacing it with “reflects,” to clarify that gradient position is interpreted as reflecting, rather than determining, individual differences in observed reappraisal success.

*The authors provide a good explanation of the processes underlying gradient shifts, largely drawing from HCP data where these gradients were originally defined. However, in the context of emotion regulation, readers may still want to know which specific brain regions' activation (Regulate vs. Look) is related to gradient-1 shifts. For example, is VLPFC activation or amygdala deactivation correlated with gradient shifts?*

**Response:** The reviewer raises an important point. To examine how neural activation during emotion regulation (Regulate vs. Look) covaries with shifts along Gradient 1, we conducted a standard second-level analysis (General Linear Model, GLM) across the joint sample (n = 619) using SPM12 (<https://www.fil.ion.ucl.ac.uk/spm/software/spm12/>). For each participant, we entered the contrast image for Regulate > Look into the group-level model and included participant-specific values of the Gradient 1 shift (ΔG1) as a covariate. We then estimated positive and negative covariate contrasts at the group level, thresholded at *p* < 0.001 (uncorrected) at the voxel level and cluster-level FWE-corrected at *p* < 0.05.

The full results of this analysis are provided in the accompanying table and figure (displaying *t*-values of the covariate map: red = positive association; blue = negative association). As expected, activation in the ventrolateral prefrontal cortex (VLPFC) was positively associated with Gradient 1 shifts. In contrast, amygdala deactivation did not show a significant association with ΔG1. Note, supplemental analyses examining coactivation for shifts along Gradients 2-5 (ΔG2- ΔG5) also found no link to activation or deactivation of the amygdala at this statistical threshold.

Notably, as illustrated in the right panel of the figure, the resulting covariate *t*-map for Gradient 1 shifts closely resembles the independently derived Gradient-1 map from the Human Connectome Project (HCP) (left panel). Thus, our results suggest that greater shifts along Gradient 1 during emotion regulation are linked to increased activation in heteromodal association cortex and decreased activation in unimodal sensory regions.

**Figure S3.** *Left:* Principal Gradient 1 map derived from independent resting-state fMRI data in the Human Connectome Project (HCP), depicting the unimodal–to–heteromodal cortical hierarchy for comparison. *Right:* Covariate *t*-map showing brain regions in which task-evoked activation during emotion regulation (Regulate > Look) covaries with participant-specific Gradient 1 shifts (ΔG1), thresholded at p < 0.001 (uncorrected) at the voxel level and cluster-level FWE-corrected at p < 0.05. Warm (red) colors reflect positive associations; cool (blue) colors reflect negative associations. The results indicate that larger Gradient 1 shifts during emotion regulation are associated with greater activation in heteromodal regions and reduced activation in unimodal regions.

## **Table S4.** Significant clusters showing covariation between Gradient 1 shifts (ΔG1) and task-evoked activation (Regulate > Look).

| Brain region | Side | k | t | MNI | | |
| --- | --- | --- | --- | --- | --- | --- |
|  |  |  |  | x | y | z |
| *Positive association* |  |  |  |  |  |  |
| Dorsal medial prefrontal cortex (dmPFC), extending to the ventral lateral prefrontal cortex (vlPFC), and dorsal lateral prefrontal cortex (dlPFC) | L & R | 5463 | 8.91 | -12 | 40 | 48 |
| Temporal parietal junction (TPJ) | L | 1043 | 9.01 | -48 | -66 | 40 |
| TPJ | R | 380 | 7.53 | 54 | -62 | 34 |
| Inferior frontal gyrus (IFG) | L | 554 | 7.49 | -46 | 26 | -10 |
| Caudate | L | 91 | 6.21 | -12 | 12 | 10 |
| Caudate | R | 58 | 5.47 | 12 | 8 | 16 |
| Middle temporal gyrus (MTG) | L | 114 | 6.53 | -54 | -36 | 6 |
| Precuneus | L & R | 359 | 5.82 | 2 | -60 | 34 |
| Middle frontal gyrus (MFG) | L | 19 | 5.11 | -32 | 54 | 6 |
| *Negative association* |  |  |  |  |  |  |
| Supplementary motor cortex, extending to the postcentral gyrus, middle cingulate cortex, central operculum, and insula | L & R | 15058 | 9.28 | 42 | 2 | 10 |
| Cuneus | R | 2210 | 7.77 | 14 | -76 | 24 |
| Lingual gyrus | L | 167 | 5.35 | -12 | -56 | -6 |

*Note.* Results are reported at p < 0.001 (uncorrected) at the voxel level and FWE-corrected at p < 0.05 at the cluster level; only peak activations of clusters are reported; L left hemisphere, R right hemisphere, k cluster size in voxels, MNI Montreal Neurological Institute.

Both the table and figure are now included in the revised supplemental materials. In addition, the unthresholded t-map has been uploaded to the OSF project folder to ensure full transparency. We now reference these materials in the revised Results section, which reads as follows:

“As a sanity check, we also examined how neural activation during emotion regulation (Regulate vs. Look) covaries with shifts along Gradient 1, using a traditional univariate analysis approach. To this end, we conducted a standard second-level analysis (General Linear Model, GLM) across the joint sample (n = 619) using SPM12 (https://www.fil.ion.ucl.ac.uk/spm/software/spm12/). For each participant, we entered the contrast image for Regulate > Look into a group-level model (GLM) and included the participant-specific Gradient 1 shift (ΔG1) as a covariate. We then estimated positive and negative covariate contrasts, thresholded at p < 0.001 (uncorrected) at the voxel level and FWE-corrected at the cluster level at p < 0.05. As expected, larger Gradient 1 shifts during emotion regulation were associated with greater activation in heteromodal regions and reduced activation in unimodal regions (see Table S4 for full details, Figure S3 for a visualization, and the OSF repository for the unthresholded t-map [<https://osf.io/yk85c/>]).” (p. 14)

*Emotion reactivity is included as a covariate predicting reappraisal success and emerges as a significant predictor. Could the authors elaborate on how they interpret this? Additionally, is emotion reactivity correlated with gradient-1? If so, this could influence the interpretation of the gradient-1 findings.*

**Response:** We thank the reviewer for this constructive comment. Individuals differ dramatically in their baseline emotion reactivity, which captures the intensity of their affective responses during passive emotion generation without regulation. Prior work has shown that emotional intensity substantially modulates the implementation and effectiveness of regulatory strategies (Moodie et al., 2020; Shafir et al., 2015; Ortner et al., 2016). Conceptually, individuals who react more strongly to emotional stimuli often have greater “room” for regulation. Empirically, both emotion reactivity and regulatory success are derived from the task-based emotion ratings, which naturally introduces correlations between the two measures.

To account for this shared variance, we included emotion reactivity as a covariate to control for individual variability in baseline emotional response, aiming to probe the unique contribution of brain gradient shifts to regulatory success above and beyond emotion reactivity. Notably, shifts along Gradient 1 remained a significant predictor of individuals’ regulatory success even after controlling for individual differences in emotion reactivity. This result indicates that Gradient 1 shifts capture variance in regulatory success that is not explained simply by how strongly individuals react to negative emotional stimuli in the first place.

In addition, following the reviewer’s request, we also examined correlations between Gradient 1 shifts and emotion reactivity. Across the discovery sample (DS), replication sample (RS), and the combined sample, emotion reactivity showed only weak associations with Gradient 1 shifts (DS: *r* = 0.10, *p* = 0.04; RS: *r* = 0.05, *p* = 0.40; Combined: *r* = 0.08, *p* = 0.03). This pattern aligns with our main finding that gradient 1 shifts primarily reflect individual differences in regulatory success. Given the weak correlations between emotion reactivity and Gradient 1 shifts, and the inclusion of emotion reactivity as a covariate in the model, we believe the interpretation of the Gradient 1 results remains robust and unchanged.

References:

Moodie, C. A., Suri, G., Goerlitz, D. S., Mateen, M. A., Sheppes, G., McRae, K., ... & Gross, J. J. (2020). The neural bases of cognitive emotion regulation: The roles of strategy and intensity. Cognitive, Affective, & Behavioral Neuroscience, 20(2), 387-407.

Ortner, C. N. M., Ste Marie, M., & Corno, D. (2016). Cognitive costs of reappraisal depend on both emotional stimulus intensity and individual differences in habitual reappraisal. PloS One, 11(12), e0167253.

Shafir, R., Schwartz, N., Blechert, J., & Sheppes, G. (2015). Emotional intensity influences pre-implementation and implementation of distraction and reappraisal. Social Cognitive and Affective Neuroscience, 10(10), 1329-1337.

*In the discussion the authors state: "our findings also have implications for the neural separability hypothesis [17]." This phrasing suggests their work represents the hypothesis itself, rather than engaging with the ongoing debate. A more precise formulation would be: "our findings also have implications for the ongoing debate on the neural separability hypothesis [17]."*

**Response:** We agree and have revised the sentence on page 20 of the revised manuscript accordingly to improve clarity.

*Figure 2D is difficult to visualize. The distributions of 'Look' and 'Regulate' appear very similar, and the dashed line does not make it clear which condition shows higher similarity with the gradients. Since most emotion regulation studies focus on the contrast between 'Look' and 'Regulate,' why not show the similarity of this contrast with the gradients, or alternatively present the distribution of ΔG?*

**Response:** Following the reviewer’s suggestion, we have now updated Figure 2D by presenting the distribution of the shifts along gradients (ΔG) to better visualize the emotion regulation-related changes in large-scale brain organization along each gradient. We agree with the reviewer that this modification will improve the clarity of the figure panel. For full transparency, we still include the original visualization of Figure 2D showing the condition-specific distributions along each gradient in the revised supplemental materials (now Figure S2). Below are the revised Figure 2 and the figure legend (p. 9):

**Figure 2.** Emotion regulation-related shifts in global activation patterns along principal gradients. **A.** Participant’s condition-specific activation maps (“Look” and “Regulate”) were projected into gradient space by calculating the dot product with each canonical gradient map (Fisher z-transformed Pearson’s r), yielding two similarity scores for each gradient per participant (one per task condition). **B.** Principal gradients were derived from fMRI resting-state data from an independent cohort (Human Connectome Project, HCP) (Margulies et al., 2016), unrelated to emotion regulation or task-evoked emotional experiences. **C.** Emotion regulation-related shifts along each gradient were calculated as within-participant differences in similarity scores between projected global activation patterns for “Regulate” and “Look” conditions for each gradient (ΔG1 to ΔG5), with higher values indicating greater neural reconfiguration along a particular principal gradient. These change scores were then used as predictors of variance in task-based emotion regulation success. **D.** Distributions of emotion regulation-related shifts across gradients (ΔG1 to ΔG5). Asterisks indicate significant differences from zero along a particular gradient (Wilcoxon signed-rank tests, * p < 0.05; ** p < 0.01; *** p < 0.001; Bonferroni corrected).

*Figure S4 only shows positive neural correlates of reappraisal success. Were any regions significant as negative correlates?*

**Response:** We believe the reviewer may be referring to Table S4 in the supplemental materials, which summarizes the significant regions associated with regulatory success (rather than Figure S4, which visualized the Neurosynth decoding results for Gradients 2-5). In the original submission, we reported only regions showing positive associations between neural activation and reappraisal success in Table S4. In response to the reviewer’s comment, we have now updated Table S4 to explicitly report both positive *and* negative associations with individual differences in reappraisal success at the group level. We apologize for the oversight in the previous version! The revised Table S4 (Table S5 in the revised supplemental materials) is provided below:

## **Table S5.** Significant clusters showing covariation between participants’ regulatory success and task-evoked activation (Regulate > Look).

| Brain region | Side | k | t | MNI | | |
| --- | --- | --- | --- | --- | --- | --- |
|  |  |  |  | x | y | z |
| *Positive association* |  |  |  |  |  |  |
| Inferior frontal gyrus (IFG) † | L | 227 | 3.70 | -38 | 14 | 24 |
| IFG | L | 138 | 3.80 | -52 | 22 | 10 |
| IFG | L | 33 | 3.58 | -36 | 30 | -8 |
| IFG | R | 27 | 3.57 | 58 | 22 | 12 |
| Middle temporal gyrus (MTG) | L | 113 | 4.24 | -50 | -36 | -4 |
| MTG | R | 13 | 3.41 | 58 | -36 | -2 |
| MTG | R | 10 | 3.23 | 58 | -12 | -16 |
| Superior temporal sulcus (STS) | L | 67 | 4.14 | -58 | -64 | 20 |
| STS | R | 24 | 3.80 | 42 | -38 | 4 |
| STS | R | 117 | 3.63 | 58 | -56 | 28 |
| Superior frontal gyrus (SFG) | R | 39 | 3.73 | 12 | 28 | 52 |
| SFG | R | 29 | 3.70 | 18 | 42 | 50 |
| SFG | L | 19 | 3.59 | -14 | 32 | 42 |
| SFG | L | 48 | 3.46 | -16 | 56 | 26 |
| Temporal pole (TP) | R | 26 | 3.66 | 2 | 20 | -20 |
| Ventral medial prefrontal cortex (vmPFC) | L | 23 | 3.41 | -10 | 46 | -10 |
| *Negative association* |  |  |  |  |  |  |
| Insula † | R | 307 | 4.32 | 44 | -24 | 24 |
| Insula | R | 10 | 3.29 | 38 | -12 | -6 |
| Supplementary motor area (SMA) | L | 153 | 4.80 | -10 | -10 | 58 |
| SMA | R | 11 | 3.31 | 10 | 6 | 44 |
| Supramarginal gyrus (SMG) | L | 180 | 4.26 | -60 | -26 | 28 |
| Posterior cingulate cortex (PCC) | L | 82 | 4.31 | -16 | -34 | 38 |
| PCC | R | 32 | 3.68 | 14 | -30 | 42 |
| Postcentral gyrus | L | 51 | 4.15 | -20 | -38 | 62 |
| Postcentral gyrus | L | 19 | 3.40 | -48 | -26 | 56 |
| Central operculum | R | 91 | 4.07 | 50 | 4 | 8 |
| Central operculum | L | 87 | 3.72 | -50 | 0 | 8 |
| Premotor cortex | R | 16 | 3.62 | 56 | 6 | 32 |
| Caudate | R | 17 | 3.51 | 22 | 18 | 20 |
| Parietal operculum | L | 13 | 3.22 | -46 | -26 | 18 |

*Note.* Results are reported at a statistical threshold of *p* < 0.001 uncorrected, with a cluster threshold of 10 voxels; † indicates results significant at *p* < 0.05, FWE corrected at the cluster level; only peak activations of clusters are reported; L left hemisphere, R right hemisphere, k cluster size in voxels, MNI Montreal Neurological Institute.

On the off-chance that the reviewer was actually referring to Figure S4: our decoding analysis was conducted using **NiMARE** (Salo et al., 2022), which computes both positive and negative correlations between the input map and meta-analytic topic maps. These correlations are descriptive rather than inferential—NiMARE does not provide p-values for these associations. Positive correlations indicate that studies associated with a given cognitive term show greater activation similarity to the input map, whereas negative correlations indicate the opposite pattern. The full decoding results with both positive and negative associations were reported in Table S6, for full transparency, and we have made all group-level activation maps and canonical gradient maps available on OSF.

Reference

Salo, T., Yarkoni, T., Nichols, T. E., Poline, J. B., Bilgel, M., Bottenhorn, K. L., ... & Laird, A. R. (2022). NiMARE: neuroimaging meta-analysis research environment. NeuroLibre, 1(1), 7.

**REVIEWER 2**

*This paper examined brain activation patterns that may indicate emotion regulation. The authors examined two large fMRI datasets that employed an emotion regulation task, and they examined principal gradients based on resting state data from the Human Connectome Project. The authors identified a gradient that was associated with emotion regulation success. The association was present in both datasets, and also was associated with negative affect in daily life in a subset of participants. The approach was novel and the question is of interest to researchers in the field. There were several issues that reduced my enthusiasm for the paper.*

1. *The gradient approach was interesting and novel but it was unclear the extent that it was exploratory versus hypothesis driven. It was also unclear why this approach was chosen over other approaches that similarly consider whole brain functional hierarchies. Further, given the reverse inference approach of the neurosynth analysis, the overall impression of this was an exploratory approach that could have worked out any number of ways. Exploratory analyses have value but the presentation did not clearly communicate the extent that the approaches were pre-determined or post hoc. Were you truly agnostic about which of the gradients to expect as significant?*

**Response:** We thank the reviewer for these thoughtful comments and appreciate the opportunity to clarify the theoretical motivation, our a priori hypothesis, and the choice of our gradient-based approach. We apologize that the initial version of the manuscript did not make these points sufficiently explicit.

First, our use of the gradient framework was not exploratory but grounded in extensive theoretical and empirical precedent. We selected this approach because principal gradients provide a well-validated, reproducible scaffold of large-scale brain organization, demonstrated across an impressive array of different modalities, such as functional, structural, and diffusion imaging, and conserved across species (Fulcher et al., 2019; Margulies et al., 2016; Paquola et al., 2019; Park et al., 2021; Tong et al., 2022; Valk et al., 2020). Critically, prior work using Neurosynth decoding showed that the principal gradient differentiating unimodal from heteromodal cortices (Gradient 1)—the gradient central to our results—maps onto a hierarchy from primary sensory processing to higher-order cognitive processes such as *emotion*, language, and social cognition (Margulies et al., 2016), providing a theoretical and conceptual link to our current project. Moreover, our own previous work published in PNAS using the same methodological gradient framework further demonstrated that systematic shifts along principal gradients track individual differences in regulatory success in the domain of dietary self-control (Janet et al., 2024), providing further theoretical and empirical precedent for choosing this approach (over other approaches that consider whole-brain functional hierarchies).

Second, with respect to the reviewer’s question about hypotheses, we were not agnostic about which gradient we expected to be most relevant. Based on extensive neuroimaging evidence that reappraisal consistently recruits heteromodal association cortex (e.g., vlPFC, dlPFC, TPJ, STS; Etkin et al., 2015; Morawetz et al., 2020), and based on prior theoretical work linking these regions to higher positions along Gradient 1, we had an a priori hypothesis that emotion regulation would produce systematic shifts along Gradient 1, and that individual differences in these shifts would relate to regulatory success in our task. We have now made this prediction explicit in the revised Introduction section of the main text.

We have revised the introduction to better clarify these points and now explicitly state our a priori hypothesis in the revised introduction:

“Based on prior evidence that reappraisal engages heteromodal (association cortex) regions (Etkin et al., 2015; Morawetz et al., 2020), we predicted a regulatory role of systematic shifts along Gradient 1, which separates unimodal from heteromodal cortices.” (p. 5)

“Building on evidence that reappraisal engages heteromodal regions and on the principle that Gradient 1 captures a hierarchy from unimodal to heteromodal cortices, we hypothesized that emotion regulation would involve shifts along this principal gradient. We further predicted that these gradient shifts would track variance in regulatory success in the laboratory and reduced negative affect in daily life.” (p. 6)

Third, regarding the Neurosynth decoding analysis: our aim was not an unconstrained exploratory reverse inference. The psychological functions associated with Gradient 1 have already been well-characterized in the literature (Margulies et al., 2016). Our decoding step therefore served as a hypothesis-driven comparison, intended to test whether the psychological profile linked to this established principal gradient aligned with the profile associated with regulatory success in our dataset. We regret that this rationale was not sufficiently clear in the prior version and have revised the text accordingly, which now reads as follows:

“Because the psychological functions associated with Gradient 1 are already well characterized in the literature (Margulies et al., 2016), our decoding step served as a hypothesis-driven test of whether the established psychological profile of this principal gradient aligned with the profile associated with regulatory success in our joint data.” (p. 16)

We hope these clarifications sufficiently address the reviewer’s concerns and make the theoretical grounding and hypothesis-driven nature of our gradient analyses more explicit.

References:

Etkin, A., Büchel, C., & Gross, J. J. (2015). The neural bases of emotion regulation. Nature Reviews Neuroscience, 16(11), 693-700.

Fulcher, B. D., Murray, J. D., Zerbi, V., & Wang, X. J. (2019). Multimodal gradients across mouse cortex. Proceedings of the National Academy of Sciences, 116(10), 4689-4695.

Janet, R., Smallwood, J., Hutcherson, C. A., Plassmann, H., Mckeown, B., & Tusche, A. (2024). Body mass index–dependent shifts along large-scale gradients in human cortical organization explain dietary regulatory success. Proceedings of the National Academy of Sciences, 121(18), e2314224121.

Margulies, D. S., Ghosh, S. S., Goulas, A., Falkiewicz, M., Huntenburg, J. M., Langs, G., ... & Smallwood, J. (2016). Situating the default-mode network along a principal gradient of macroscale cortical organization. Proceedings of the National Academy of Sciences, 113(44), 12574-12579.

Morawetz, C., Riedel, M. C., Salo, T., Berboth, S., Eickhoff, S. B., Laird, A. R., & Kohn, N. (2020). Multiple large-scale neural networks underlying emotion regulation. Neuroscience & Biobehavioral Reviews, 116, 382-395.

Park, B. Y., De Wael, R. V., Paquola, C., Larivière, S., Benkarim, O., Royer, J., ... & Bernhardt, B. C. (2021). Signal diffusion along connectome gradients and inter-hub routing differentially contribute to dynamic human brain function. Neuroimage, 224, 117429.

Paquola, C., Vos De Wael, R., Wagstyl, K., Bethlehem, R. A., Hong, S. J., Seidlitz, J., ... & Bernhardt, B. C. (2019). Microstructural and functional gradients are increasingly dissociated in transmodal cortices. PLoS Biology, 17(5), e3000284.

Tong, C., Liu, C., Zhang, K., Bo, B., Xia, Y., Yang, H., ... & Liang, Z. (2022). Multimodal analysis demonstrating the shaping of functional gradients in the marmoset brain. Nature Communications, 13(1), 6584.

Valk, S. L., Xu, T., Margulies, D. S., Masouleh, S. K., Paquola, C., Goulas, A., ... & Eickhoff, S. B. (2020). Shaping brain structure: Genetic and phylogenetic axes of macroscale organization of cortical thickness. Science Advances, 6(39), eabb3417.

2. *The neurosynth exploration for theory-neutral explanations seems at odds with the premise of the neural systems topographical landscape. The coordinates reflect task based activations that survive multiple threshold corrections, etc, but do not reflect a whole brain assessment of how the brain accomplishes the tasks associated with the coordinates. This approach seems to contradict the premise of the rest of the paper—that the whole brain could contribute in hierarchical ways.*

**Response:** We thank the reviewer for raising this important point regarding the use of Neurosynth decoding. To clarify, our decoding analyses were conducted on *unthresholded, whole-brain* maps for both the gradient maps and the regulatory-success covariate map. We intentionally chose this approach to align with the core premise of the manuscript—namely, that hierarchical functional organization is a *whole-brain* property rather than something that can be captured by thresholded, task-specific peak activations alone.

We fully agree with the reviewer that functional hierarchy involves distributed processing across the entire brain, as reflected most prominently along Gradient 1 (the unimodal–heteromodal axis) examined in this work. By applying Neurosynth decoding to unthresholded whole-brain maps, we aimed to characterize the psychological functions associated with these *global* activation patterns rather than isolated clusters. This approach therefore provides a theory-neutral framework that is fully consistent with—and directly supports—the manuscript’s whole-brain perspective on functional gradients and regulatory success. We thank the reviewer for giving us a chance to clarify this issue. The revised manuscript further clarifies this issue:

“Importantly, using Neurosynth to decode unthresholded whole-brain maps allowed us to characterize the psychological functions reflected in distributed activation patterns rather than isolated clusters, offering an interpretive framework that complements the manuscript’s whole-brain perspective.” (p. 16)

3. *The conclusions seem to overstate the results. "These findings advance a network-level account of regulatory success, offering a biologically grounded, ecologically valid framework for understanding adaptive emotional functioning" This seems to imply that previous imaging approaches were not biologically grounded? Or were not ecologically valid? I did not find compelling evidence presented to reach those same conclusions. Also, the "network level account" seems to overstate the approach—the network function was not probed independently of the task to determine the extent that it contributed to emotion function. It seemed context dependent. Activation patterns within a network framework seems necessary to establish the network level conclusion, but not sufficient. It seems that further probing would be required to rule out alternative explanations.*

**Response:** We thank the reviewer for raising this thoughtful concern about the interpretation of our conclusions. We did not intend to suggest that prior neuroimaging work on emotion regulation is *not* biologically grounded or ecologically valid—indeed, these approaches have been foundational to the field. Our aim was instead to articulate the advantages of situating emotion regulation within the *principal gradient framework*.

Our use of the term “biologically grounded” refers to the fact that the principal gradients represent a well-validated, cross-modal, and cross-species organizational scaffold of the brain. These gradients are reproducible across functional, structural, and diffusion modalities of the brain (Margulies et al., 2016; Paquola et al., 2019; Park et al., 2021; Valk et al., 2020) and are conserved across species, such as rodents and non-human primates (Fulcher et al., 2019; Tong et al., 2022). Leveraging this gradient framework allows us to locate emotion-regulation processes within an independently established, biologically meaningful hierarchy of large-scale brain organization. This complements—rather than critiques—traditional region- and network-based approaches that have substantially advanced our understanding of regulatory mechanisms.

Similarly, our reference to “ecologically valid” was meant to highlight that Gradient 1 shifts were related not only to in-scanner regulatory success, but also to affective experiences assessed through daily experience sampling in daily life. The convergence between laboratory-based and real-world indices underscores the ecological relevance of the gradient-derived neurobiomarkers, without implying that earlier studies lacked ecological validity.

To further clarify this point, we have revised the sentence in the abstract, which now reads as follows:

“These findings introduce a gradient-based perspective on emotion regulation success that is biologically grounded in well-established large-scale brain organization and ecologically valid through its links with real-world emotional experience.” (p. 2)

We also appreciate the reviewer’s point regarding contextual dependence. To clarify, the principal gradients used in our analyses were derived from the independent HCP sample based on resting-state data, reflecting intrinsic organization rather than task-evoked emotional or regulatory processes. We then projected task-evoked activation patterns onto this *pre-existing* large-scale hierarchy to examine how regulatory processes align with intrinsic organization. In this sense, the task did not define network structure; rather, it was interpreted within an established organizational scaffold.

Finally, we agree that our findings demonstrate associations between individual differences in regulatory success and large-scale brain organization, rather than establishing causal network involvement. Future work combining gradient methods with neuromodulation (e.g., TMS/tDCS) or lesion models will be important for testing whether modulating specific network states directly influences regulatory outcomes, which we addressed in the discussion:

“These gradient-based markers may also prove useful in tracking treatment response to interventions such as regulation training, psychotherapy, neurostimulation, or neurofeedback (Bewernick et al., 2010; Kadosh et al., 2016; Linhartová et al., 2019; Moses et al., 2023). Moreover, future work integrating gradient approaches with neuromodulation techniques (e.g., TMS/tDCS) or lesion models may offer causal leverage, allowing researchers to test whether directly manipulating specific network states can alter regulatory outcomes.” (p. 23)

References:

Bewernick, B.H., et al., Nucleus accumbens deep brain stimulation decreases ratings of depression and anxiety in treatment-resistant depression. Biological psychiatry, 2010. 67(2): p. 110-116.

Fulcher, B. D., Murray, J. D., Zerbi, V., & Wang, X. J. (2019). Multimodal gradients across mouse cortex. Proceedings of the National Academy of Sciences, 116(10), 4689-4695.

Kadosh, K.C., et al., Using real-time fMRI to influence effective connectivity in the developing emotion regulation network. Neuroimage, 2016. 125: p. 616-626.

Linhartová, P., et al., fMRI neurofeedback in emotion regulation: A literature review. NeuroImage, 2019. 193: p. 75-92.

Margulies, D. S., Ghosh, S. S., Goulas, A., Falkiewicz, M., Huntenburg, J. M., Langs, G., ... & Smallwood, J. (2016). Situating the default-mode network along a principal gradient of macroscale cortical organization. Proceedings of the National Academy of Sciences, 113(44), 12574-12579.

Moses, T.E., et al., Effects of neuromodulation on cognitive and emotional responses to psychosocial stressors in healthy humans. Neurobiology of Stress, 2023. 22: p. 100515.

Park, B. Y., De Wael, R. V., Paquola, C., Larivière, S., Benkarim, O., Royer, J., ... & Bernhardt, B. C. (2021). Signal diffusion along connectome gradients and inter-hub routing differentially contribute to dynamic human brain function. Neuroimage, 224, 117429.

Paquola, C., Vos De Wael, R., Wagstyl, K., Bethlehem, R. A., Hong, S. J., Seidlitz, J., ... & Bernhardt, B. C. (2019). Microstructural and functional gradients are increasingly dissociated in transmodal cortices. PLoS Biology, 17(5), e3000284.

Tong, C., Liu, C., Zhang, K., Bo, B., Xia, Y., Yang, H., ... & Liang, Z. (2022). Multimodal analysis demonstrating the shaping of functional gradients in the marmoset brain. Nature Communications, 13(1), 6584.

Valk, S. L., Xu, T., Margulies, D. S., Masouleh, S. K., Paquola, C., Goulas, A., ... & Eickhoff, S. B. (2020). Shaping brain structure: Genetic and phylogenetic axes of macroscale organization of cortical thickness. Science Advances, 6(39), eabb3417.

**REVIEWER 3**

*The authors projected global fMRI activation patterns from an emotion regulation (ER) lab task onto the principal sensory-transmodal gradient (G1) from the HCP datasets. In both datasets, individual differences in ER success were predicted by systematic differences along G1. Gradient based reconfiguration also predicted lower negative affect in daily life as measured with a smartphone app. Neurosynth meta-analysis revealed that G1 and ER success align with social cognition, memory, attention, and negative emotions. The results are promising indicators that gradient based coordinate systems may help to better understand the network basis of emotional and regulatory processes in the brain, and could be informative for the design of brain based biomarkers.*

*This is overall a well written and well structured paper. Methods appear state-of-the-art and the study features an independent replication as well as generalization to smartphone real life behavioral prediction. I have the following somewhat minor comments for the authors to improve on their already accomplished work:*

**Response:** We appreciate the reviewer’s positive assessment of our work. We have addressed each comment in detail below and believe that the resulting revisions have further strengthened the manuscript.

*1) In the introduction, the authors may want to briefly discuss some conceptual and empirical work from Lisa Feldman Barrett's group that connected gradient research with emotional and allostatic processes (https://pmc.ncbi.nlm.nih.gov/articles/PMC11117115/). Other work that related gradients to information processing hierarchies in the human brain, in both healthy and diseased populations, may be relevant as it can help to dissociate more sensory from higher order integrative and regulatory processes.*

**Response:** We thank the reviewer for bringing these references to our attention. We have revised our literature review on information processing hierarchies in the human brain and recent work from Lisa Feldman Barrett's group that connected gradient research with emotional and allostatic processes in the introduction, which now read as follows:

“Principal gradients track a hierarchy from primary sensory processing to high-order functions such as emotion, language, and social cognition, offering a structural scaffold for hierarchical information processing (Huntenburg et al., 2018; Margulies et al., 2016). Multimodal neuroimaging work shows that gradients capture functional diversity across cognitive tasks, underscoring the role of large-scale brain organization in shaping brain function (Wang et al., 2025). Evidence from epilepsy demonstrates that gradients sensitively track reorganization of hippocampal and neocortical systems during episodic memory states (Cabalo et al., 2024). Alterations in large-scale brain organization and hierarchy are also observed in autism, accounting for diverse symptomology and neurodevelopmental features (Bernhardt et al., 2025; Hong et al., 2019; Park et al., 2021). The gradient framework further aligns with theoretical accounts of allostasis, which propose that the brain predicts and regulates internal bodily states through coordinated, whole-brain organization (Katsumi et al., 2022; Katsumi et al., 2023). Within this view, hierarchical gradients may support domain-general neural functions, offering insight into how emotional processing and other cognitive phenomena may reflect whole-brain, allostatic computations rather than distinct, localized processes.” (p. 4)

References:

Bernhardt, B. C., Valk, S. L., Hong, S. J., Soulières, I., & Mottron, L. (2025). Autism-related shifts in the brain’s information processing hierarchy. Trends in Cognitive Sciences.

Cabalo, D. G., DeKraker, J., Royer, J., Xie, K., Tavakol, S., Rodríguez-Cruces, R., ... & Bernhardt, B. C. (2024). Differential reorganization of episodic and semantic memory systems in epilepsy-related mesiotemporal pathology. Brain, 147(11), 3918-3932.

Hong, S. J., Vos de Wael, R., Bethlehem, R. A., Lariviere, S., Paquola, C., Valk, S. L., ... & Bernhardt, B. C. (2019). Atypical functional connectome hierarchy in autism. Nature Communications, 10(1), 1022.

Huntenburg, J. M., Bazin, P. L., & Margulies, D. S. (2018). Large-scale gradients in human cortical organization. Trends in Cognitive Sciences, 22(1), 21-31.

Katsumi, Y., Theriault, J. E., Quigley, K. S., & Barrett, L. F. (2022). Allostasis as a core feature of hierarchical gradients in the human brain. Network Neuroscience, 6(4), 1010-1031.

Katsumi, Y., Zhang, J., Chen, D., Kamona, N., Bunce, J. G., Hutchinson, J. B., ... & Barrett, L. F. (2023). Correspondence of functional connectivity gradients across human isocortex, cerebellum, and hippocampus. Communications Biology, 6(1), 401.

Margulies, D. S., Ghosh, S. S., Goulas, A., Falkiewicz, M., Huntenburg, J. M., Langs, G., ... & Smallwood, J. (2016). Situating the default-mode network along a principal gradient of macroscale cortical organization. Proceedings of the National Academy of Sciences, 113(44), 12574-12579.

Park, B. Y., Hong, S. J., Valk, S. L., Paquola, C., Benkarim, O., Bethlehem, R. A., ... & Bernhardt, B. C. (2021). Differences in subcortico-cortical interactions identified from connectome and microcircuit models in autism. Nature Communications, 12(1), 2225.

Wang, Y., Eichert, N., Paquola, C., Rodriguez-Cruces, R., DeKraker, J., Royer, J., ... & Bernhardt, B. C. (2025). Multimodal gradients unify local and global cortical organization. Nature Communications, 16(1), 3911.

*2) Page 8 - the authors suddenly mention 5 gradients. It might be worthwhile to also describe these 5 gradients a bit more in the introduction and also the results, as not everyone may have an intuitive understanding of how they partition variance across the cortica; surface (eg in terms of sensory-transmodal, visual-somatomotor, multiple demand, etc)…They kinda do this on P.9, so there could be earlier mention of them I find.*

**Response:** Thank you for drawing our attention to this issue. We now introduce the five principal gradients in the revised Introduction:

“These five dimensions of brain variation differentiate unimodal and heteromodal (association) cortices (Gradient 1), visual and sensorimotor cortices (Gradient 2), the default mode network (DMN) and frontoparietal systems (FP) (Gradient 3), ventral and dorsal attention networks (Gradient 4), and visual cortex and ventral attention networks (Gradient 5) (Figure 2B).” (p. 5)

Moreover, we have now also added the description of the gradients and a preamble at the beginning of the gradient-related portion of the Results section before diving into the specific results. Below is the updated text:

“Specifically, we examined task-evoked activation patterns within the first five principal gradients derived from an independent HCP dataset, which together explain ~55% of variance and differentiate major functional systems—unimodal and heteromodal association cortices (Gradient 1), visual and sensorimotor cortices (Gradient 2), default mode network and frontoparietal systems (Gradient 3), ventral and dorsal attention networks (Gradient 4), and visual cortex and ventral attention network (Gradient 5) (Figure 2B).” (p. 8)

*3) Can the authors clarify in the methods/findings whether findings were derived from cortical data only, or whether cortical and subcortical data were analyzed together. If the former, a brief justification may help and/or a demonstration of consistency when all GM voxels are analyzed/mapped.*

**Response:** The reviewer raises an interesting point. Our main gradient findings were derived from whole-brain gray matter voxels (cortical and subcortical), as emotion regulation engages distributed networks spanning both cortical and subcortical systems. Thus, analyzing cortical and subcortical data jointly was theoretically motivated by extensive prior work on neural substrates of emotion regulation. To avoid confusion, we have clarified our analytical approach in the revised Methods section, which now reads as follows:

“Gradient maps encompassed both cortical and subcortical regions, reflecting the involvement of distributed neural networks of cortical and subcortical areas in emotion processing [11, 18, 52, 65].” (p. 28)

“Notably, all computations were based on whole-brain principal gradient maps that included both cortical and subcortical voxels.” (p. 28)

In direct response to the reviewer’s question, we also conducted supplementary analyses using cortical data only. Importantly, the core results remain qualitatively unchanged, further supporting the robustness of our approach. For brevity, we have not included these supplemental analyses in the revised manuscript, but we would be happy to incorporate them if the reviewer or editorial team believes they would benefit PLOS Biology readers.

*4) Its mainly semantics, but I would refrain from using the term 'predict' when reporting the LMM findings. For within sample correlations, 'Explain' or 'Correlate' is possibly more accurate but they can further emphasize the DS to RS prediction in the findings and abstract.*

**Response:** We thank the reviewer for these suggestions. We agree that the original phrasing using “predict” was inappropriate for the within-sample analysis. Accordingly, for the within-sample LMM findings, we have replaced “predict” with non-causal language such as “associated with” to ensure accurate interpretation. Following the reviewer’s suggestion, we have retained the term “predict” when describing the out-of-sample/cross-sample prediction analyses.

*5) Is neurovault still a thing? They could deposit the unthresholded maps there as well in addition to osf.*

**Response:** Following the reviewer’s suggestion, we have now uploaded the unthresholded maps to Neurovault (https://neurovault.org/collections/22324/) in addition to OSF (<https://osf.io/yk85c/>).

*6) This final point is more exploratory, but after reading the discussion, I wondered whether similar findings could emerge if the analyses were confined to a specific region previously implicated in emotion regulation and emotional reactivity—such as the amygdala—rather than using whole-cortex data. For example, one could derive region-specific gradients and test whether shifts in activity within that space correlate with ER success and predict individual differences. If such effects do not emerge at the regional level, it could further support the idea that large-scale, cortex-wide organization is critical for understanding emotion regulation.*

**Response:** The reviewer raises an intriguing point. To directly assess the relationship between gradient shifts and regulatory success at the regional level, we conducted an additional analysis in which we extracted region-specific gradient shifts from both cortical and subcortical areas and examined their relationships with variance in regulatory success. For cortical regions, we used the Schaefer-400 cortical parcellation, consistent with prior region-specific gradient studies (Areshenkoff et al., 2024; Zhu et al., 2025). We used the Harvard-Oxford Subcortical Atlas to define subcortical regions. We then estimated linear mixed models (LMMs) in which region-specific gradient shifts served as predictor variables of individual regulatory success, controlling for age, sex assigned at birth, and emotional reactivity, and with study ID included as a random effect. Because these models were repeated across all regions, we applied false discovery rate (FDR) correction separately for cortical (400 regions) and subcortical (14 regions) sets.

Interestingly, no cortical or subcortical region showed a significant association with variance in regulatory success after FDR correction (q = 0.05). This suggests that the observed relationship between gradient shifts and regulatory success is not driven by isolated regional effects. Instead, these findings further support the interpretation that emotion regulation induces systematic, large-scale changes in activation across distributed brain regions, and that this gradient-based reconfiguration reliably reflects individual differences in regulatory success.

We have now incorporated this additional analysis into our revised Methods and Results sections, which read as follows:

“Fifth, to further assess whether large-scale gradient shifts, rather than effects confined to specific regions, account for individual differences in regulatory success, we conducted a supplemental region-wise analysis. We extracted region-specific gradient shifts from both cortical and subcortical areas and examined their associations with individual differences in task-based regulatory success. For the cortical analysis, we used the Schaefer-400 parcellation, consistent with prior region-specific gradient work (Areshenkoff et al., 2024; Zhu et al., 2025), and we used the Harvard–Oxford Subcortical Atlas (Makris et al., 2006) to define 14 subcortical regions. We then estimated a linear mixed model (LMM) in which region-specific gradient shifts were entered as predictors of individual regulatory success, adjusting for age, sex assigned at birth, and emotional reactivity, and including study ID as a random effect. Because this model was repeated across all regions, we applied a false discovery rate (FDR) correction to account for multiple comparisons, performing the correction separately for cortical (400 regions) and subcortical (14 regions) regions.” (p. 32)

“Region-wise Gradient Shifts Do Not Correspond to Variance in Regulatory Success. As a validation analysis, we assessed whether regional (cortical and subcortical) gradient shifts were related to regulatory success. After FDR correction (q = 0.05), no individual regions showed significant associations with task-based regulatory success scores, suggesting that the whole-brain gradient effect is not driven by isolated regional sources.” (p. 19)

References:

Areshenkoff, C. N., de Brouwer, A. J., Gale, D. J., Nashed, J. Y., Smallwood, J., Flanagan, J. R., & Gallivan, J. P. (2024). Distinct patterns of connectivity with the motor cortex reflect different components of sensorimotor learning. PLoS Biology, 22(12), e3002934.

Makris, N., Goldstein, J. M., Kennedy, D., Hodge, S. M., Caviness, V. S., Faraone, S. V., ... & Seidman, L. J. (2006). Decreased volume of left and total anterior insular lobule in schizophrenia. Schizophrenia Research, 83(2-3), 155-171.

Zhu, T., Areshenkoff, C. N., De Brouwer, A. J., Nashed, J. Y., Flanagan, J. R., & Gallivan, J. P. (2025). Contractions in human cerebellar-cortical manifold structure underlie motor reinforcement learning. Journal of Neuroscience, 45(18).
