# Supplementary material for: Emotion regulation success involves systematic gradient-based reconfigurations of large-scale activation patterns in the human brain
Source: PLoS Biol. 2026 Apr 2;24(4):e3003666. doi: 10.1371/journal.pbio.3003666 (PMC13046165; doi:10.1371/journal.pbio.3003666)
Supplement: Attachment — Submitted filename: response_letter_4submission_auresp_3.docx [file pbio.3003666.s022.docx]

**Response Letter**

Dear Editors and Reviewers,

We sincerely appreciate your careful evaluation of our revised manuscript and are delighted to hear that it is likely to be accepted for publication at PLOS Biology. We are grateful to the editors and reviewers for the time and effort devoted to assessing our work.

In response to the editorial requests, we have revised the manuscript title, added links to the relevant funding agencies and the ethics approval numbers, and verified the completeness of the data and code deposited on the Open Science Framework (OSF), ensuring full compliance with PLOS Biology’s data and code policies.

Thank you again for considering our manuscript.

Sincerely,

Anita Tusche, Carmen Morawetz, Remi Janet, and Ruien Wang

**EDITOR COMMENTS**

*Dear Dr Wang,*

*Thank you for your patience while we considered your revised manuscript "Emotion Regulation in the Gradient Framework: Large-Scale Brain Organization Reflects Individual Differences in Reappraisal Success" for publication as a Research Article at PLOS Biology. This revised version of your manuscript has been evaluated by the PLOS Biology editors, the Academic Editor and two the original reviewers.*

*Based on the reviews, we are likely to accept this manuscript for publication, provided you satisfactorily address the following data and other policy-related requests:*

** We would like to suggest a different title to improve its accessibility for our broad audience:*

*Emotion regulation involves systematic gradient-based reconfigurations of large-scale activation patterns in the human brain*

** Please add the links to the funding agencies in the Financial Disclosure statement in the manuscript details.*

** Please include the approval/license numbers of the ethical approval for the experiments.*

** DATA POLICY:*

*You may be aware of the PLOS Data Policy, which requires that all data be made available without restriction: http://journals.plos.org/plosbiology/s/data-availability. For more information, please also see this editorial: http://dx.doi.org/10.1371/journal.pbio.1001797*

*Note that we do not require all raw data. Rather, we ask that all individual quantitative observations that underlie the data summarized in the figures and results of your paper be made available in one of the following forms:*

*1) Supplementary files (e.g., excel). Please ensure that all data files are uploaded as 'Supporting Information' and are invariably referred to (in the manuscript, figure legends, and the Description field when uploading your files) using the following format verbatim: S1 Data, S2 Data, etc. Multiple panels of a single or even several figures can be included as multiple sheets in one excel file that is saved using exactly the following convention: S1_Data.xlsx (using an underscore).*

*2) Deposition in a publicly available repository. Please also provide the accession code or a reviewer link so that we may view your data before publication.*

*Regardless of the method selected, please ensure that you provide the individual numerical values that underlie the summary data displayed in the figure panels as they are essential for readers to assess your analysis and to reproduce it.*

*NOTE: the numerical data provided should include all replicates AND the way in which the plotted mean and errors were derived (it should not present only the mean/average values).*

*Please also ensure that figure legends in your manuscript include information on where the underlying data can be found, and ensure your supplemental data file/s has a legend.*

*Please ensure that your Data Statement in the submission system accurately describes where your data can be found.*

** CODE POLICY*

*Per journal policy, if you have generated any custom code during the course of this investigation, please make it available without restrictions. Please ensure that the code is sufficiently well documented and reusable, and that your Data Statement in the Editorial Manager submission system accurately describes where your code can be found. More information on our Code Policy, what and how to share can be found here: https://journals.plos.org/plosbiology/s/code-availability*

*Please note that we cannot accept sole deposition of code in GitHub, as this could be changed after publication. However, you can archive this version of your publicly available GitHub code to Zenodo. Once you do this, it will generate a DOI number, which you will need to provide in the Data Accessibility Statement (you are welcome to also provide the GitHub access information). See the process for doing this here:* [*https://docs.github.com/en/repositories/archiving-a-github-repository/referencing-and-citing-content*](https://docs.github.com/en/repositories/archiving-a-github-repository/referencing-and-citing-content)

*As you address these items, please take this last chance to review your reference list to ensure that it is complete and correct. If you have cited papers that have been retracted, please include the rationale for doing so in the manuscript text, or remove these references and replace them with relevant current references. Any changes to the reference list should be mentioned in the cover letter that accompanies your revised manuscript.*

*In addition to these revisions, you may need to complete some formatting changes, which you will receive in a follow up email. A member of our team will be in touch with a set of requests shortly. If you do not receive a separate email within a few days, please assume that checks have been completed, and no additional changes are required.*

*We expect to receive your revised manuscript within two weeks.*

*To submit your revision, please go to https://www.editorialmanager.com/pbiology/ and log in as an Author. Click the link labelled 'Submissions Needing Revision' to find your submission record. Your revised submission must include the following:*

*- a cover letter that should detail your responses to any editorial requests, if applicable, and whether changes have been made to the reference list*

*- a Response to Reviewers file that provides a detailed response to the reviewers' comments (if applicable, if not applicable please do not delete your existing 'Response to Reviewers' file.)*

*- a track-changes file indicating any changes that you have made to the manuscript.*

*NOTE: If Supporting Information files are included with your article, note that these are not copyedited and will be published as they are submitted. Please ensure that these files are legible and of high quality (at least 300 dpi) in an easily accessible file format. For this reason, please be aware that any references listed in an SI file will not be indexed. For more information, see our Supporting Information guidelines:*

*https://journals.plos.org/plosbiology/s/supporting-information*

**Published Peer Review History**

*Please note that you may have the opportunity to make the peer review history publicly available. The record will include editor decision letters (with reviews) and your responses to reviewer comments. If eligible, we will contact you to opt in or out. Please see here for more details:*

*https://plos.org/published-peer-review-history/*

**Press**

*Should you, your institution's press office or the journal office choose to press release your paper, please ensure you have opted out of Early Article Posting on the submission form. We ask that you notify us as soon as possible if you or your institution is planning to press release the article.*

**Protocols deposition**

*To enhance the reproducibility of your results, we recommend that if applicable you deposit your laboratory protocols in protocols.io, where a protocol can be assigned its own identifier (DOI) such that it can be cited independently in the future. Additionally, PLOS ONE offers an option for publishing peer-reviewed Lab Protocol articles, which describe protocols hosted on protocols.io. Read more information on sharing protocols at https://plos.org/protocols?utm_medium=editorial-email&utm_source=authorletters&utm_campaign=protocols*

*Please do not hesitate to contact me should you have any questions.*

*Sincerely,*

*Christian*

*Christian Schnell, PhD*

*Senior Editor*

*cschnell@plos.org*

*PLOS Biology*

**EDITORIAL REQUESTS**

** We would like to suggest a different title to improve its accessibility for our broad audience:*

*Emotion regulation involves systematic gradient-based reconfigurations of large-scale activation patterns in the human brain*

**Response:** We thank the editors for the thoughtful suggestion regarding the title and agree that the proposed version is more accessible to a broader readership. Given that the present study places a strong emphasis on individual differences in emotion regulation success, we would like to propose a slight modification based on the editors’ suggestion. The revised title would read:

“Emotion regulation success involves systematic gradient-based reconfigurations of large-scale activation patterns in the human brain”

** Please add the links to the funding agencies in the Financial Disclosure statement in the manuscript details.*

**Response:** We have now added links to the funding agencies in the revised Financial Disclosure Section.

** Please include the approval/license numbers of the ethical approval for the experiments.*

**Response**: We have now included all the study-specific ethic board and approval numbers information in Table S10 in the revised supplementary materials. Please note that for Studies 2–6 in the replication sample, the local ethics boards did not provide approval/licence numbers for the approved protocols. However, ethics approval was obtained from the local ethics boards listed in the revised Table S10, and we are happy to provide the documentation to the editors if needed.

** DATA POLICY*

**Response:** We can confirm that all individual observations used in the main analysis and figure generation have been deposited in OSF (<https://osf.io/yk85c/>), which is public and accessible to anyone with the link. Regarding the raw fMRI data, the data for discovery sample is publicly available on Neurovault (<https://neurovault.org/collections/16266/>); for replication sample, the data for study 7 is available on OSF, <https://osf.io/yk85c/>; studies 1-6 are available upon request. The unthresholded covariate maps are also available on OSF (<https://osf.io/yk85c/>) and on Neurovault (<https://neurovault.org/collections/22324/>). We can also confirm that all of the above information is clearly described in the Code and Data Availability section.

In addition, we have revised the figure legends in both the main text and the supplementary materials to include links to the underlying data.

** CODE POLICY*

**Response:** We can confirm that all custom code from this work have been made publicly available in the OSF repository (<https://osf.io/yk85c/>). And this information is clearly described in the Code and Data Availability section.

** Reference List*

**Response:** We can confirm that the current reference list is complete and correct.

**REVIEWER COMMENTS**

**Reviewer #2:**

*Thank you for the thoughtful attention to all the issues raised previously. This version reads better and seems like a valuable contribution.*

**Response:** We sincerely appreciate the time and effort the reviewer put into the review process and are grateful for the valuable comments and feedback.

**Reviewer #3:**

*The authors have been very responsive to all the reviews and the paper is now ready for publication.*

**Response:** We sincerely appreciate the reviewer’s time and effort in reviewing our manuscript and thank the reviewer for their careful and thorough evaluation.
